# Analyses of nervous system patterning genes in the tardigrade *Hypsibius exemplaris* illuminate the evolution of panarthropod brains

**DOI:** 10.1186/s13227-018-0106-1

**Published:** 2018-07-30

**Authors:** Frank W. Smith, Mandy Cumming, Bob Goldstein

**Affiliations:** 1 Biology Department, University of North Florida, Jacksonville, FL USA; 2 Biology Department, University of North Carolina at Chapel Hill, Chapel Hill, NC USA

**Keywords:** Brain evolution, Nervous system evolution, Body plan evolution, Homology

## Abstract

**Background:**

Both euarthropods and vertebrates have tripartite brains. Several orthologous genes are expressed in similar regionalized patterns during brain development in both vertebrates and euarthropods. These similarities have been used to support direct homology of the tripartite brains of vertebrates and euarthropods. If the tripartite brains of vertebrates and euarthropods are homologous, then one would expect other taxa to share this structure. More generally, examination of other taxa can help in tracing the evolutionary history of brain structures. Tardigrades are an interesting lineage on which to test this hypothesis because they are closely related to euarthropods, and whether they have a tripartite brain or unipartite brain has recently been a focus of debate.

**Results:**

We tested this hypothesis by analyzing the expression patterns of *six3*, *orthodenticle*, *pax6*, *unplugged*, and *pax2/5/8* during brain development in the tardigrade *Hypsibius exemplaris*—formerly misidentified as *Hypsibius dujardini*. These genes were expressed in a staggered anteroposterior order in *H. exemplaris*, similar to what has been reported for mice and flies. However, only *six3*, *orthodenticle*, and *pax6* were expressed in the developing brain. *Unplugged* was expressed broadly throughout the trunk and posterior head, before the appearance of the nervous system. *Pax2/5/8* was expressed in the developing central and peripheral nervous system in the trunk.

**Conclusion:**

Our results buttress the conclusion of our previous study of Hox genes—that the brain of tardigrades is only homologous to the protocerebrum of euarthropods. They support a model based on fossil evidence that the last common ancestor of tardigrades and euarthropods possessed a unipartite brain. Our results are inconsistent with the hypothesis that the tripartite brain of euarthropods is directly homologous to the tripartite brain of vertebrates.

**Electronic supplementary material:**

The online version of this article (10.1186/s13227-018-0106-1) contains supplementary material, which is available to authorized users.

## Background

How brains evolved is one of the most perplexing questions in biology. Recent debates have centered on how to interpret similarities in brain development of distantly related animals [1–9]. During brain development in mice, *Otx2* is expressed in the forebrain and midbrain. A paralogous group of paired box genes, *Pax2*, *Pax5*, and *Pax8*, along with the homeobox gene *Gbx2*, exhibit strong expression near the midbrain–hindbrain boundary. In the hindbrain and more posterior regions of the developing central nervous system, Hox genes are expressed [10]. Orthologs of these genes are expressed in a similar staggered anteroposterior pattern in the tripartite brain of flies [10]. Based on these correspondences, it has been hypothesized that the ancient ancestor of mice and flies—the last common ancestor of Nephrozoa [11]—had a tripartite brain [2, 10, 12–15].

Similarities extend to specific regions of the brains of protostomes and deuterostomes. *Six3* orthologs are expressed in the anteriormost region of the developing brain in representatives of both protostomes and deuterostomes—a region that gives rise to neurosecretory cells in both lineages [16]. *Pax6* is expressed in a lateral region of the brain in many protostomes and deuterostomes that have been investigated [17–19]. Structural and developmental similarities between the vertebrate pallium and annelid mushroom bodies [20], and between the vertebrate basal ganglia and the arthropod central complex [21] have also been identified. These similarities add support to the model of a nephrozoan ancestor with a complex brain [2, 3, 7, 9, 10, 14].

Objections against this view of brain evolution have been raised [4, 5, 8, 22]. Each main lineage of bilaterians includes representatives that lack complex brains, and several bilaterian lineages are characterized by diffuse nervous systems, rather than centralized nervous systems [23]. Cladistic analyses suggest that the nephrozoan ancestor exhibited a nerve net and that centralized nervous systems evolved independently between five and nine times [4, 5], while brains evolved up to four times independently [8]. Intriguingly, orthologs of the genes that pattern the tripartite brains of flies and mice exhibit regionalized expression patterns during development of the hemichordate *Saccoglossus kowalevskii*, even though hemichordates lack a brain, and instead exhibit a much more diffuse nervous system [24–26]. Likewise, genes that pattern tripartite brains show similar regionalized expression patterns in molluscs that exhibit diffuse nervous systems [27–31]. Therefore, genes that control development of the tripartite brains of insects and vertebrates exhibit conserved regionalized expression patterns in animals with diffuse nervous systems—animals that are predicted to have inherited their diffuse nervous systems from the last common ancestor of Nephrozoa [4, 5]. If so, this would indicate that these genes were independently co-opted for the evolution of tripartite brains several times in the nephrozoan lineage [1, 24, 25].

**Fig. 1:**
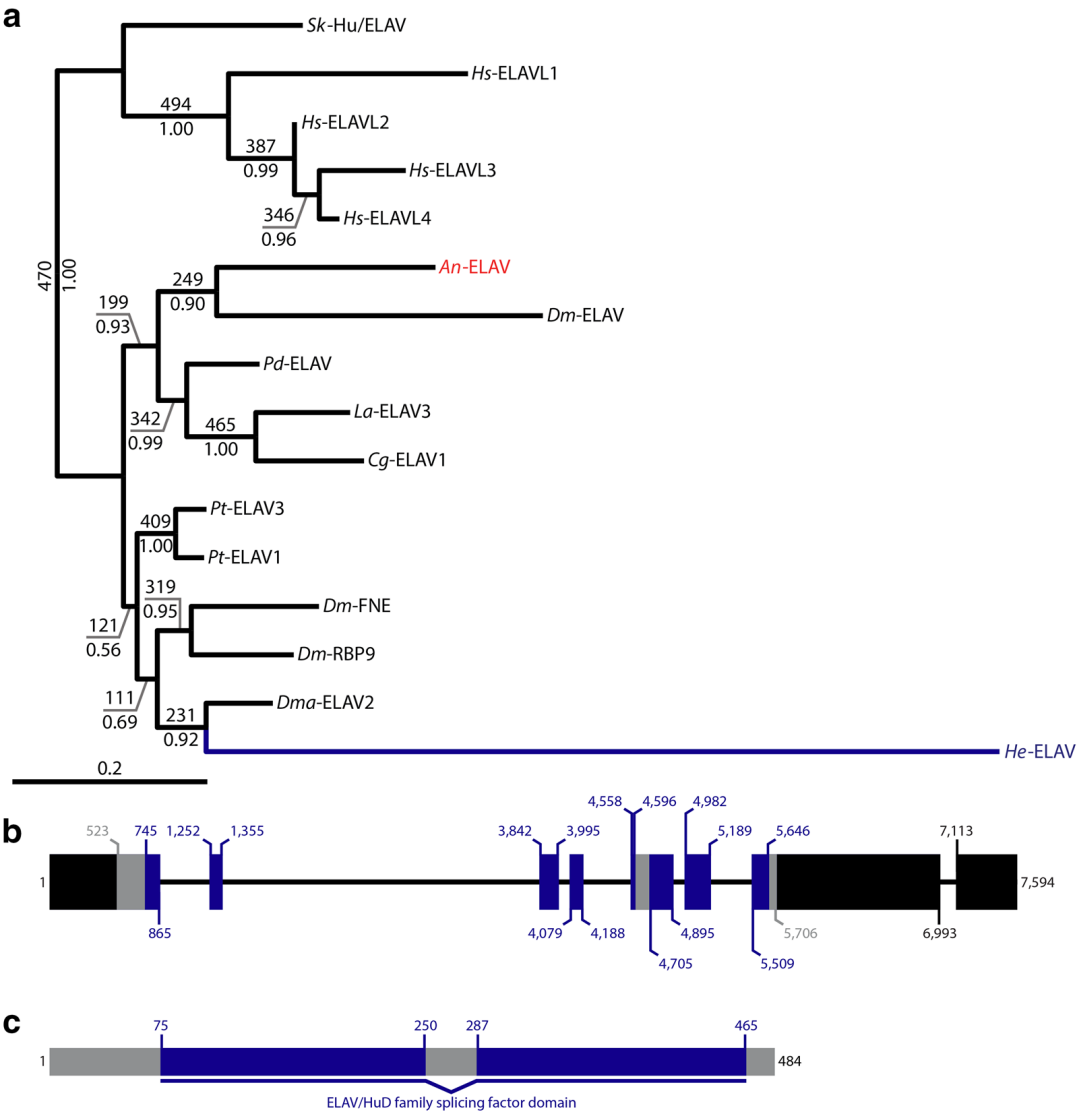
Phylogenetic and structural analyses of *He*-*elav*. **a** ELAV phylogeny. Maximum likelihood tree topology is shown. Bootstrap support values (out of 500 replicates) are shown above branches and posterior probabilities are shown below branches. The branch leading to *He*-ELAV and its name are colored dark blue. The name of An-ELAV is colored red. **b** Genomic structure of *He*-*elav*. Relative to the sense strand, 5′ is to the left. Thick bars represent exons. Thin bars represent introns. Black = UTR. Blue = sequence that codes for the ELAV/HuD family splicing domain. Gray = all other coding sequences. The numbers represent nucleotide positions (see Additional file 1: Table S1). **c** Structure of *He*-ELAV protein. The *N*-terminus is to the left. Blue = ELAV/HuD family splicing domain. Gray = all other protein sequences. The numbers represent amino acid positions (see Additional file 1: Table S1). *An*, anonymous sequence (see main text); Cg, *Crassostrea gigas*; *Dm*, *Drosophila melanogaster*; *Dma*, *Daphnia magna*; *He*, *Hypsibius exemplaris*; *Hs*, *Homo sapiens*; *Pd*, *Platynereis dumerilii*; *Sk*, *Saccoglossus kowalevskii*

To trace the evolutionary history of complex nervous systems in Nephrozoa, additional taxa from diverse metazoan lineages must be investigated [1, 32, 33]. Studies of tardigrades may help elucidate nervous system evolution. Tardigrada is closely related to Euarthropoda [34–36] and, like euarthropods, tardigrades exhibit a segmented centralized nervous system (Fig. 1a) [37–39]. Several paired lobes are recognizable in the tardigrade brain, which have been interpreted as homologs of the proto-, deuto-, and tritocerebral brain segments of insects and other euarthropods [40–43]. By contrast, our recent analysis of Hox genes in the tardigrade *Hypsibius exemplaris*—formerly misidentified as *H. dujardini* and renamed *H. exemplaris* to reflect its burgeoning status as a model system [44]—suggested that the brain of this species is homologous to just the protocerebrum of euarthropods [45] and therefore exhibits unipartite morphology. Distinguishing between these hypotheses has important implications for our understanding of the evolution of tripartite brain morphology.

To test the homology of the tardigrade brain to the brains of other animals, we investigated the expression patterns of several genes in *H. exemplaris* that have been implicated in the development of brains in both vertebrates and insects. Our results build on our analysis of Hox genes, demonstrating that tardigrades have a unipartite brain. These results support a model in which euarthropods evolved a tripartite brain after they diverged from tardigrades.

## Methods

### Maintaining *H. exemplaris* cultures

We used a standard protocol to culture *H. exemplaris* [46].

### β-Tubulin immunostaining

To visualize the developing nervous system, we stained *H. exemplaris* embryos with a β-tubulin antibody (E7, Developmental Studies Hybridoma Bank). To do this, we modified a method that was successfully implemented to stain the nervous system of hatchling [38] and adult *H. exemplaris* specimens [47] using the β-tubulin antibody, a method used to detect antibody localization in *H. exemplaris* embryos [48], and a method for embryonic in situ hybridization for this species [45]. Staged embryos were washed for 1 h in a permeabilization buffer (5 units chitinase (Sigma-Aldrich C6137), 10 mg chymotrypsin (Sigma-Aldrich C4129), 1 ml 50 mM potassium phosphate buffer (pH 6.0)), followed by three 5-minute washes in 0.5X PBTw (0.5X phosphate-buffered saline, 0.1% Tween-20, pH 7.4). Embryos were fixed in 4% formaldehyde/33% heptane in 0.5X PBTw for 30 min at RT. Embryos were then washed five times for 5 min with 0.5X PBTw. Next, embryos were taken through a MeOH dilution series, consisting of 25, 50, 70, and 90% MeOH in 0.5X PBTw, followed by three washes in 100% MeOH. At this stage, embryos were kept in a − 20 °C freezer for at least 20 min. (Embryos can be stored indefinitely at this point.) Embryos were then taken through a reverse dilution series of MeOH and washed three times with 0.5X PBTw. Embryos were cut out of their eggshells with a 25-gauge needle and then washed three times for 10 min and four times for 30 min in 0.2% bovine serum albumin in 0.5X PBTw (BSA). Next, they were washed two times for 30 min in 5% normal goat serum in 0.5X PBTw (NGS). Embryos were then incubated overnight in a 1:100 dilution of the β-tubulin antibody in 5% NGS at 4 °C. The next day, embryos were washed three times for 5 min and four times for 30 min in 0.5X PBTw. This was followed by two 30-minute washes in NGS and an overnight wash in a 1:200 dilution of a goat anti-mouse Cy3-conjugated secondary antibody (Jackson ImmunoResearch) in NGS. The following day, embryos were washed three times for 5 min and six times for 30 min with 0.5X PBTw.

### Identifying candidate genes and phylogenetic analyses

We performed reciprocal BLAST searches using human and *D. melanogaster* sequences as queries to identify candidate genes from *H. exemplaris*. Initial analyses focused on our draft genome assembly [49]. Three studies identified containment sequences in our draft assembly for *H*. *exemplaris* [50–52]. Therefore, candidate genes that we identified with this method were then used as queries in BLAST search analyses of three independent transcriptome data sets [52–54] and two independent genome assemblies for *H. exemplaris* [51, 52], to verify that they were true *H. exemplaris* sequences. For phylogenetic analyses, we aligned sequences using MUSCLE [55]. We trimmed our matrix using Gblocks [56]. We performed both Bayesian and maximum likelihood analyses using the LG model [57] with an estimated proportion of invariable sites and an estimated gamma shape parameter with four substitution rate categories. We used PhyML for maximum likelihood analyses [58], with branch supports calculated by 500 bootstrap replicates. We used MrBayes for Bayesian analyses [59]. The Bayesian analyses ran for 450,000 generations after the standard deviation of split frequencies dropped below 0.01, with trees sampled every 100 generations. Posterior probabilities were calculated from 4500 trees from the posterior tree distribution. We identified domains in the predicted protein sequences of candidate *H. exemplaris* orthologs with CD-Search [60]. We identified intron/exon boundaries by comparing transcriptome sequences to genome sequences using Splign [61]. When we could not find an ortholog of interest in *H. exemplaris* data sets, we tested for presence of the ortholog in the genome of *Ramazzottius varieornatus* [62] using reciprocal BLAST searches.

### Cloning

Genes of interest were amplified with PCR from *H. exemplaris* embryonic cDNA, or from *H. exemplaris* genomic DNA in the case of *He*-*pax6*. Primers used for this study were as follows: *He*-*elav*, 5′-GCATCCAGAACAAGAACATCAAGG-3′, 5′-ACTGGGAAAAGCGAAGTGTCTAGC-3′; *He*-*otd*, 5′-GTTCCCGCACCGAGGAAACAG-3′, 5′-CTCTCACGTCCTCCACGCTGA-3′; *He*-*pax2/5/8*, 5′-CGTTTTCCTTCAGACTTTCGTCGT-3′, 5′-TCCGATAACTCGTCTCGTTTCCTC-3′; *He*-*pax6*, 5′-CGTTTTATTTGCACACAGCGAGATA-3′, 5′-ATCTACCGGATTGCAAAGTTCTGG-3′; *He*-*six3*, 5′-ATCTTCACTTGACGCGATTGTGGT-3′, 5′-GTCCTTGCTGTTATCCTCGTCCAT-3′; *He*-*unplugged* (*unpg*), 5′-TTGCGAGAGAAACAAAACTGGATG-3′, 5′-CAAAACAAACGCGCCAAGTG-3′; *An*-*elav* outer, 5′-AAACGATGACACAAGACGAAATTA-3′, 5′-CAGCATACCTGTACCTATTCATGG-3′; *An*-*elav* inner, 5′-GTTCAGTTGGTGCTATTGAGTCAT-3′, 5′-AGGTAGATAGGATGGTGCTAATGG-3′; *An*-*pax2/5/8* outer, 5′-TATAAACATTTGGTGGAGACGACA-3′, 5′-GAATCAATCAACTTGGAGGAGTTT-3′; *An*-*pax2/5/8* inner, 5′-ATGGTGAGTGCGACTATCATCTTCG-3′, 5′-ATGGTGAGTGCGACTATCATCTTC-3′, 5′-ACATCAGTCGACAGTTACGAGTGT-3′; *An*-*poxm* outer, 5′-CGATTTGCATAACGTAACGTACTC-3′, 5′-GAACTGGAAAGAAATGATCGAACT-3′; *An*-*poxm* inner, 5′-GCAGACTTTTATTGATGTTGTTCG-3′, 5′-TTTCAAAGTGATTCAAACCAAGAA-3′. Genes were cloned into the pCR™4-TOPO^®^ (Invitrogen) vector following the manufacturers protocol. Minipreps for each clone were made using a QIAprep Spin Miniprep Kit (Qiagen) and following the manufacturers protocol. Miniprep plasmids were sequenced to verify their identity.

### In situ hybridization

To make in situ probes, we linearized plasmids with PCR by using the M13 forward and reverse primers that come with the TOPO™ TA Cloning™ Kit for Sequencing (Invitrogen). Linearized template was used in a transcription reaction using either T7 or T3 RNA polymerase (Promega), DIG RNA labeling mix (Roche), and following the manufacturer’s protocols. Synthesized probes were cleaned using an RNAeasy^®^ Mini Kit and following the manufacturer’s instructions. In situ hybridization was performed as previously described [45]. For counter staining the nervous system of in situ hybridization specimens, the β-tubulin antibody was added after we washed out the anti-DIG-AP (Roche) antibody. Instead of washing out the anti-DIG-AP antibody with maleic acid buffer, as we normally do for in situ hybridizations, for counter staining with the β-tubulin antibody, we washed it out with 0.5X PBTw. After these washes, we followed the β-tubulin immunostaining method described above. After completion of the immunostaining procedure, we washed the embryos with maleic acid buffer (pH 9.5) and continued through the development steps of the in situ hybridization method.

### Imaging

After in situ hybridization and/or β-tubulin immunostaining, embryos were mounted in DAPI Fluoromount-G (SouthernBiotech). Fluorescent images were collected on a Zeiss 710 LSM. Maximum projections were produced in ImageJ. DIC images were taken on a Nikon Eclipse 800 microscope. Minimum and maximum displayed pixel values were adjusted in ImageJ and/or Photoshop. In cases where the in situ stain occluded β-tubulin or DAPI signal, we used either the Cyan Hot or glow LUT in ImageJ to better visualize the β-tubulin or DAPI signal.

## Results

### Identification of an *elav* ortholog

ELAV/HuD family splicing factors are RNA-binding proteins that are almost exclusively expressed in differentiating and mature neurons [63]. We used *elav* expression to identify early stages in nervous system development in *H. exemplaris* embryos. We identified two candidate *elav* genes in our *H. exemplaris* nucleotide data. One of these predicted genes was present in all data sets that we analyzed, except for the Yoshida et al. [52] transcriptome (Additional file 1: Table S1). We refer to this sequence as *He*-*elav*. The other candidate *elav* ortholog was missing in all other data sets that we analyzed except for our *H. exemplaris* genome assembly (Additional file 2: Table S2) [49]. We were unable to amplify this gene from genomic DNA using nested PCR (Additional file 3: Fig. S1). Furthermore, this predicted gene sequence is located on a small scaffold of only 7049 nucleotides in our genome assembly and it is the only gene predicted to be located on this scaffold. Therefore, we concluded that this sequence most likely represents a contaminant in our genome assembly. We call this sequence anonymous *elav* (*An*-ELAV), referring to its likely inclusion as a contaminant from an unknown organism.

We performed phylogenetic analyses on a matrix of 239 amino acid positions from 14 ELAV sequences stemming from 10 species, in addition to *He*-ELAV and *An*-ELAV (Additional file 1: Table S1; Additional file 2: Table S2; Additional file 4: FASTA alignments). Both of these sequences were nested within a well-supported clade (470/500 bootstrap support; 1.0 posterior probability) that separated all deuterostome *elav* genes from all protostome *elav* genes (Fig. 1a). We interpret this as phylogenetic support for the assignment of both sequences to the *elav* orthology group. A CD-Search detected an ELAV/HuD family splicing factor domain (Accession TIGR1661; E-value = 6.88e-123) characteristic of ELAV proteins in the *He*-*elav* sequence that we identified (Fig. 1b, c). Interestingly, 37 amino acids within the ELAV/HuD family splicing factor domain were not recognized as part of this domain by the CD-Search algorithm (between amino acid positions 250 and 287, Fig. 1c). We also detected the nucleotide sequence that is predicted to give rise to this intervening region in the *He*-*elav* sequence that we amplified from embryonic cDNA.

### Expression pattern of *He*-*elav* and β-tubulin

**Fig. 2:**
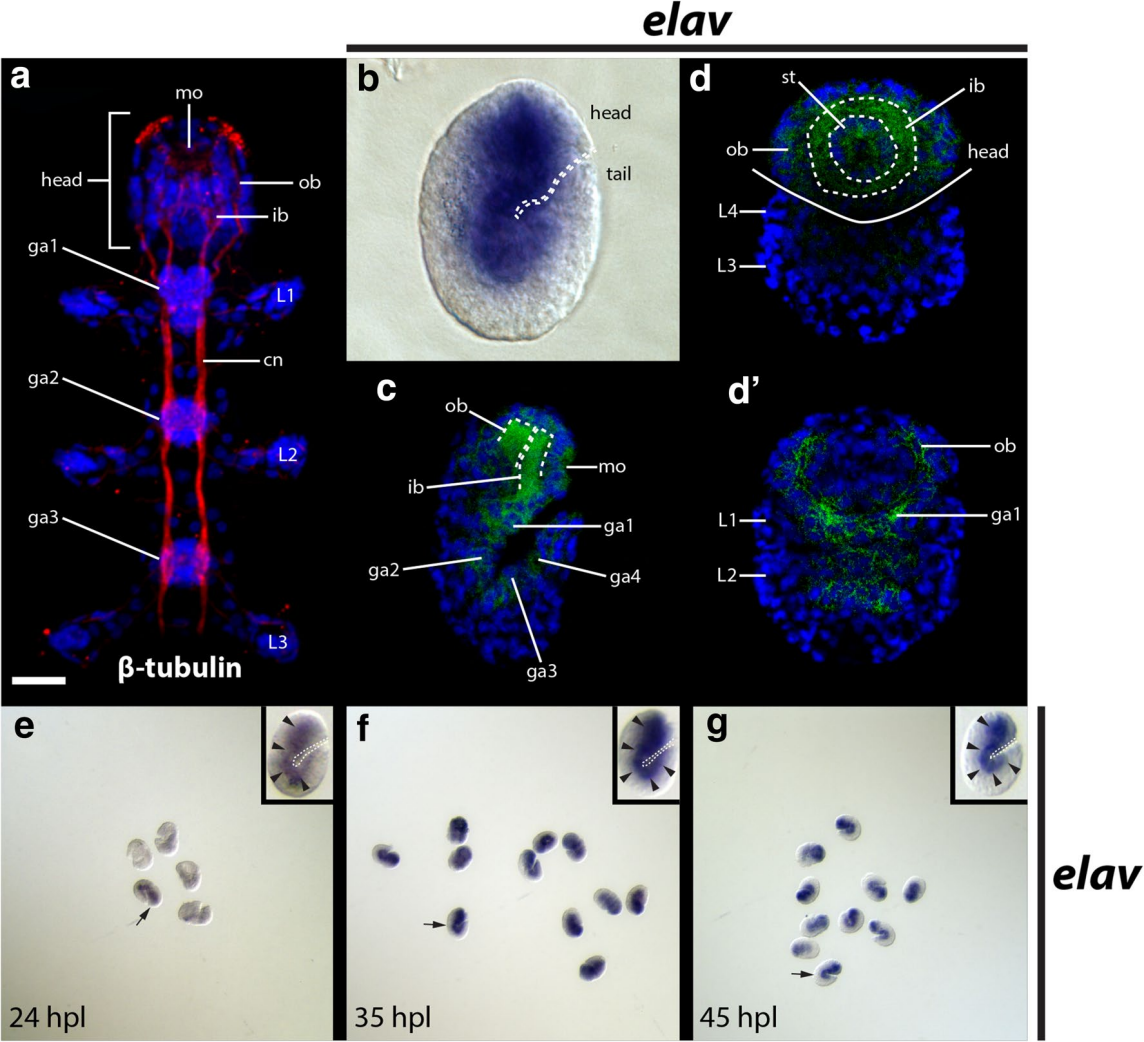
Embryonic expression of *He*-*elav*. **a**, **c**, **d** Nuclei are stained with DAPI (blue). **a** Maximum projection of a *H. exemplaris* hatchling showing the central nervous system. The head and first three trunk segments are shown. Neurons are stained with a fluorescent secondary antibody bound to a β-tubulin antibody (red). The scale bar in the bottom left corner of the top panel equals 10 μm. **b** DIC image of an in situ hybridization targeting *He*-*elav* (blue) in a laterally mounted 45 hpl embryo. The dashed line traces the ventral surface of the embryo. **c**, **d** Confocal micrographs of *He*-*elav* (green) embryos in 45 hpl embryos. **c** Maximum projection of a laterally mounted embryo. Dashed lines trace the inner and outer brain regions. **d**, **d**′ Individual slices from a *Z*-series showing a frontal view of the head of an embryo. **d**′ A deeper slice than (**d**). The solid line in (**d**) traces the ventral part of the head. The dashed lines trace the inner brain space where neuropil and commissures are found in fully developed brains. **e**–**g** Results of in situ hybridizations targeting *He*-*elav* at different developmental stages. Arrows point to specimens that are enlarged (inset). In the inset panels, all specimens are laterally mounted, facing right, with anterior toward the top. Arrowheads in the inset panels point to *He*-*elav* expression in the developing brain and ventral nerve cord. Dashed lines trace the ventral surface of the specimens in the inset panels. cn, connective; ga1–ga4, ganglion 1–ganglion 4; hpl, hours post-laying; ib, inner brain region; L1–L4, leg 1–leg 4; mo, mouth; ob, outer brain region; st, stomodeal complex

We used a β-tubulin antibody to visualize the nervous system of *H. exemplaris*. The central nervous system of *H. exemplaris* includes a brain that is housed in the head and ventral trunk nervous system [47]. Inner connectives (ic) extend from the inner brain region (ib) to the anteriormost trunk ganglion (ga1; Fig. 2a) [47]. The inner brain region is composed of neuropil [64]. Outer connectives extend between the outer brain region (ob) and the anteriormost trunk ganglion (Fig. 2a) [47]. Neurites of the inner and outer brain regions meet at a thick band of dorsal neuropil [64]. Trunk ganglia adjoin adjacent trunk ganglia by paired connectives (cn) [47]. We detected this basic nervous system architecture with the β-tubulin antibody in 45 h post-laying (hpl) embryos (Additional file 5: Movie 1). The nervous system of our 45 hpl embryos appears to be in the final stage of development, based on a staging series for *H. exemplaris* [65].

For in situ hybridizations, we focused on three developmental stages: 24 hpl, when evidence of ectodermal segmentation is apparent, but the nervous system and legs are not; 35 hpl, when developing ganglia are apparent, and leg buds are discernible; and 45 hpl, when legs have reached their final size and the central nervous system exhibits the connectivity that is visible post-embryonically (Additional file 5: Movie 1) [38]. Strong *He*-*elav* staining in 45 hpl embryos was restricted to the region where the central nervous system develops (Fig. 2b–d). The *He*-*elav* in situ pattern closely matched the pattern of β-tubulin localization in 45 hpl embryos (compare Fig. 2b–d to Additional file 5: Movie 1). At 24 hpl, expression of *He*-*elav* staining was noticeably variable between specimens (Fig. 2e). Only one specimen exhibited *He*-*elav* staining near the future position of the central nervous system (black arrowhead, Fig. 2e), and *He*-*elav* staining was relatively light in this specimen compared to 35 hpl and 45 hpl embryos (Fig. 2f, g). This indicates that specification of neuronal identity begins in embryos at 24 hpl or shortly after.

### Identification of a *six3* ortholog

**Fig. 3:**
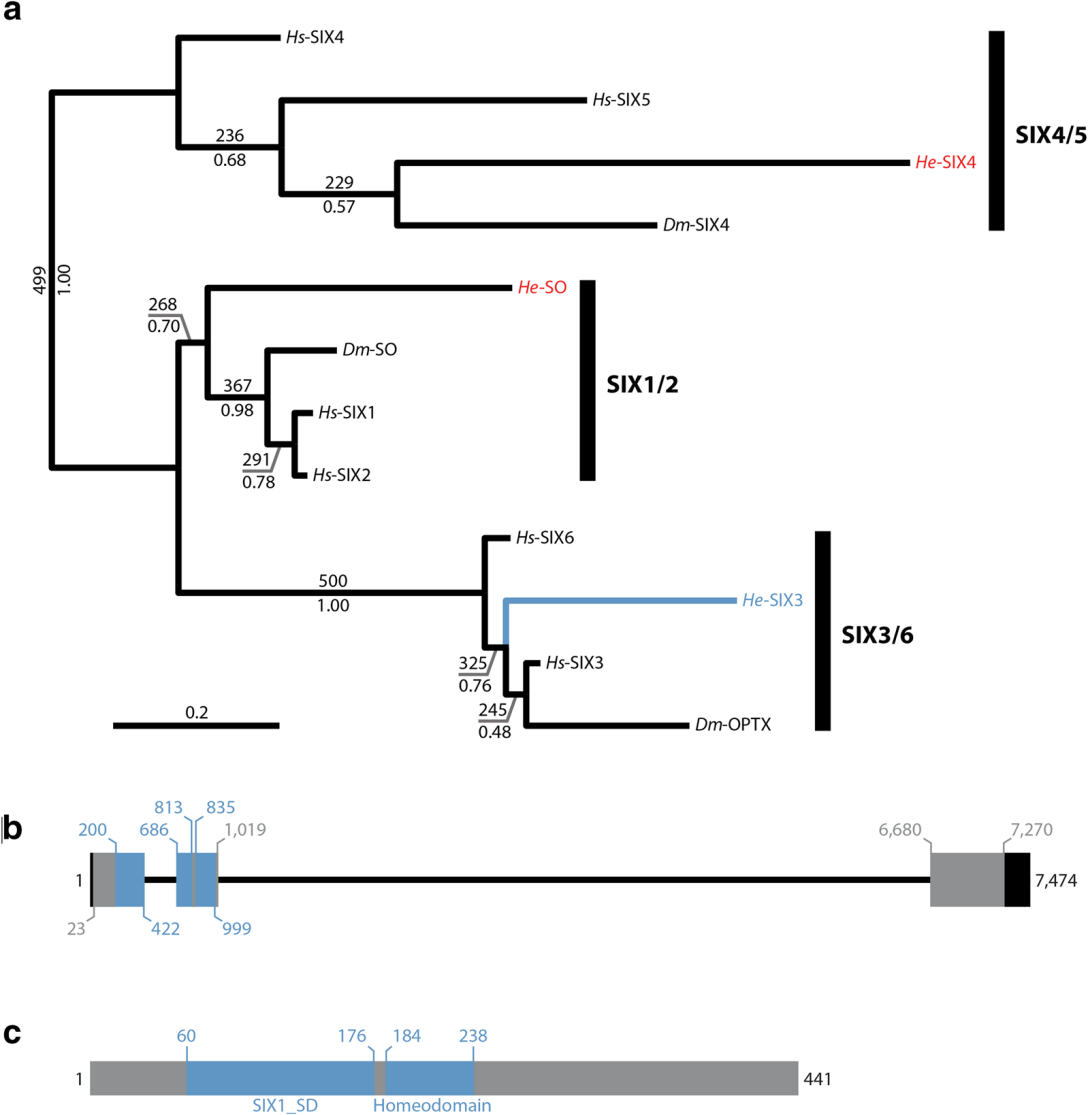
Phylogenetic and structural analyses of *He*-*six3*. **a** Phylogeny of Six family protein sequences. Tree topology resulted from a maximum likelihood analysis. Bootstrap support values are shown above branches (out of 500 bootstrap replicates). Posterior probabilities, based on a Bayesian analysis, are shown below branches. The branch leading to *He*-Six3 and its name are colored light blue. The names of other *H. exemplaris* proteins in the tree are colored red. **b** Structure of the *He*-*six3* gene in the genome. Relative to the sense strand, 5′ is to the left. Thick lines represent exons. Black = UTR. Light blue = sequence that codes for conserved domains. Gray = all other coding sequences. Thin black lines represent introns. The numbers represent nucleotide positions (see Additional file 1: Table S1). **c** Structure of the *He*-SIX3 protein. The N-terminus is to the left. Regions that are predicted to be conserved protein domains are colored blue. All other protein sequences are colored gray. The numbers represent amino acid positions (see Additional file 1: Table S1). *Dm, Drosophila melanogaster*; *He*, *Hypsibius exemplaris*; *Hs*, *Homo sapiens*

The Six family includes three groups of genes—*Six1/2*/*sine oculis*, *Six3/6*/*optix*, and *Six4/5* [66]. We identified three-candidate Six family genes in available *H. exemplaris* genome and transcriptome assemblies (Additional file 1: Table S1; Additional file 2: Table S2). We performed a phylogenetic analysis using a matrix that included 164 amino acid positions (Additional file 4: FASTA alignments). Each candidate gene from *H. exemplaris* fell within a different Six family group in our phylogenetic analysis. If we rooted the tree with the candidate gene that appeared to be nested within the *Six3/6*/*optix* group, this group was no longer monophyletic. This suggests that this candidate gene represents an *H. exemplaris* ortholog of the *Six3/6*/*optix* group. This gene contains a predicted SIX domain (SIX1_SD) and a homeodomain (Fig. 3b, c), both of which are characteristics of Six family genes [66]. Because the direct ortholog of *Six3*/*optix* is referred to as *six3* in most animals, we refer to the *H. exemplaris* ortholog as *He*-*six3.*

### Expression pattern of *He*-*six3*

**Fig. 4:**
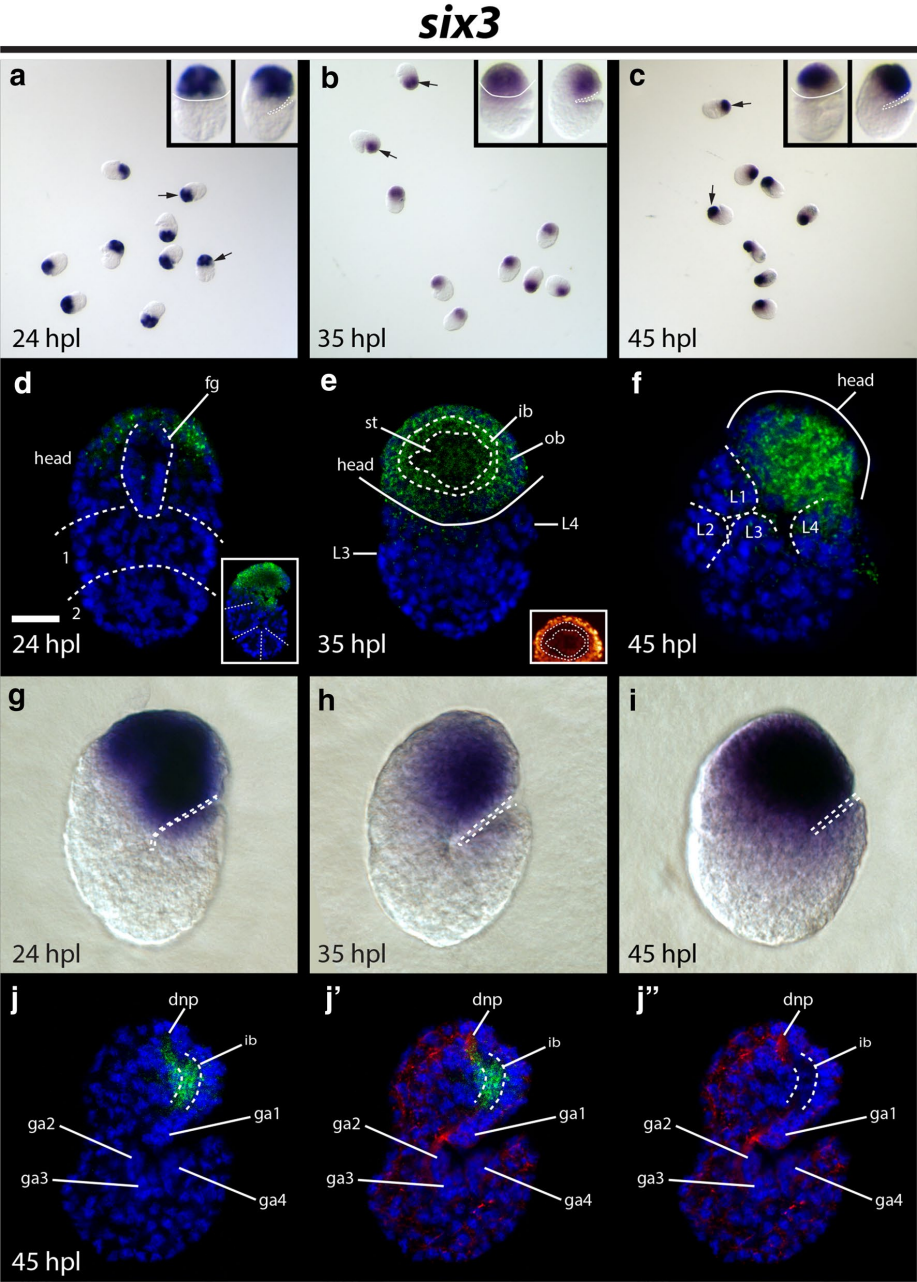
Embryonic *He*-*six3 expression.* **a**–**c** Fields of *He*-*six3-*stained embryos at 24 hpl, 35 hpl, and 45 hpl, respectively. Arrows point to specimens that are enlarged in the inset panels. **a**–**c** (Inset) The left inset specimens in panels (**a**, **b**) are facing forward, with anterior toward the top. The left inset specimen in panel (**c**) is facing forward, but rotated slightly left. Solid white lines trace the space between the anterior and posterior ends of the specimens. The right inset specimens in panels (**a**–**c**) are laterally mounted, facing right, with anterior toward the top; a dashed line traces the ventral surface. **d**–**f**, **j**–**j**″ Confocal micrographs. *He*-*six3* expression is shown in green. DAPI labels nuclei (blue). **d** Dorsal view of a 24 hpl embryo. Dashed lines trace the boundaries between the head and trunk segment 1, and between trunk segments 1 and 2. A dashed line also outlines the developing foregut. The scale bar equals 10 μm. (Inset) A laterally mounted 24 hpl embryo; dashed lines trace segment boundaries. **e** Frontal view—relative to the head—of a 35 hpl embryo. The solid line traces the ventral surface of the head. Dashed lines outline the inner brain space where neuropil and commissures are located in the fully developed brain. (Inset) The head of the same specimen using the glow LUT in ImageJ to help visualize nuclei. **f** Oblique view of a 45 hpl embryo. The specimen is laterally mounted, and facing right, but slighted rotated onto its back. Legs on the right side of the body are outlined. **g**–**i** DIC images of in situ hybridizations. **g**, **h** Laterally mounted embryos. A dashed line traces the ventral surface. **i** Oblique view of a 45 hpl embryo. The specimen is positioned as in (**f**), but is a different specimen. A dash line traces the ventral surface of the head and the ventral surface of the posteriormost leg of the right side of the body. **j**–**j**″ A laterally mounted 45 hpl embryo. The lateral part of the inner brain space, where neuropil is found in fully developed brains, is outlined. **j**′–**j**″ β-tubulin expression is colored red. dnp, dorsal neuropil; fg, foregut; ga1–ga4, ganglion 1–ganglion 4; hpl, hours post-laying; ib, inner brain region; L1–L4, leg 1–leg 4; ob, outer brain region; st, stomodeal complex

Strong *He*-*six3* signal was detected during all developmental periods that we investigated (Fig. 4a–c). At 24 hpl, *He*-*six3* signal was detected broadly across the ectoderm in the developing head (Fig. 4d, g). *He*-*six3* signal did not extend to the posterior border of the dorsal head at 24 hpl (Fig. 4d). At 35 hpl, *He*-*six3* transcripts were detected in regions of the head that will give rise to both the outer (ob) and inner (ib) brain regions (Fig. e, h). We also detected transcripts near the developing stomodeal complex in 35 hpl embryos (st; Fig. 4e). At 45 hpl, *He*-*six3* signal was detected broadly across the lateral ectoderm of the head (Fig. 4f). We also detected *He*-*six3* signal in the region of the inner brain where neuropil develops, but not in the dorsal neuropil (dnp; compare Fig. 4j to frames 19–27 of Additional file 5: Movie 1).

### Identification of an *otd* ortholog

**Fig. 5:**
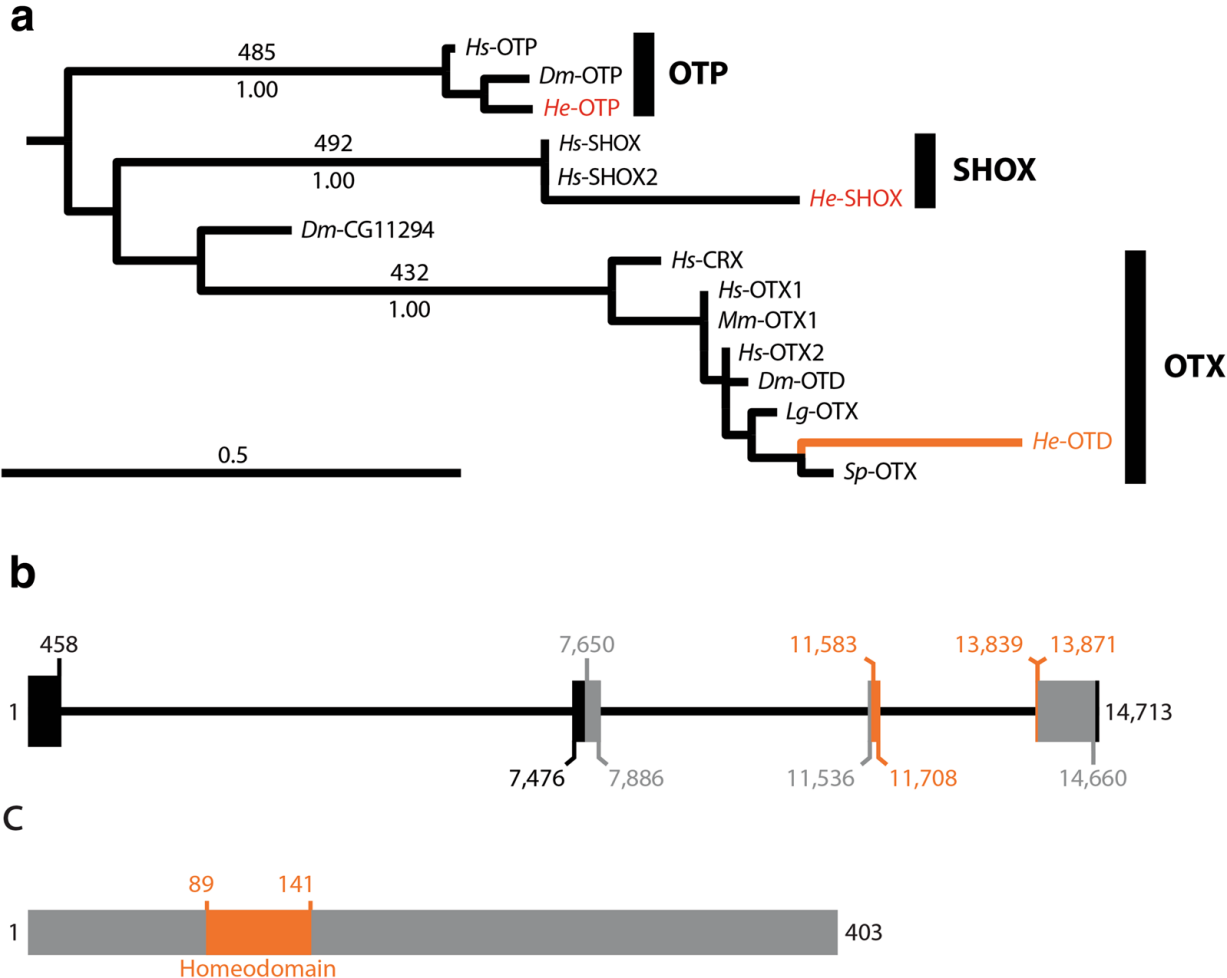
Phylogenetic and structural analyses of *He*-*otd*. **a** A phylogeny of PRD class homeobox genes based on analyses of the homeodomain. The full PRD class phylogeny is shown in Additional file 6: Fig. S2. The names of different PRD families—based on Ryan et al. [67]—are shown in large bold text. Maximum likelihood tree topology is shown. Bootstrap support values, based on 500 replicates, are shown above branches. Posterior probabilities, based on a Bayesian analysis, are shown below branches. The branch leading to *He*-OTD and its name are colored orange. The names of other PRD class homeobox genes from *H. exemplaris* are colored red. The OTX clade (boxed) is enlarged in A′. **b** Structure of the *He*-*otd* gene in the genome. Relative to the sense strand, 5′ is to the left. Thick lines represent exonic regions. Black = UTR. Orange = position of sequence that codes for the homeodomain. Gray = all other coding sequence. Thin black lines represent intronic regions. The numbers represent nucleotide positions (see Additional file 1: Table S1). **c** Structure of the *He*-OTD protein. The N-terminus is to the left. The region that corresponds to the homeodomain is colored orange. All other protein sequences are colored gray. Numbers represent amino acid positions (see Additional file 1: Table S1). *Dm, Drosophila melanogaster*; *He*, *Hypsibius exemplaris*; *Hs*, *Homo sapiens*; *Lottia gigantea*; *Mm*, *Mus musculus*; *Sp, Strongylocentrotus purpuratus*

We previously identified an *otd* ortholog in *H. exemplaris* [45]. In order to better characterize this gene from a phylogenetic perspective, we performed an analysis that included *He*-*otd* and candidate *H. exemplaris* PRD Class homeobox genes from our genome assembly (Additional file 1: Table S1; Additional file 2: Table S2). We followed a previously published naming scheme for PRD Class homeobox families [67]. Our matrix included the 60 amino acid long homeodomains of 74 PRD Class homeobox proteins stemming from several species (Additional file 4: FASTA alignments). In the maximum likelihood tree, *He*-OTD was nested within a highly supported monophyletic OTX clade (Fig. 5a; 432/500, bootstrap support; 1.00 Bayesian posterior probability; Additional file 6: Fig. S2). The homeodomain was the only conserved domain present in the predicted *He*-OTD protein (Fig. 5b, c), as is the case for *D. melanogaster* OTD. We did not detect Pax3/7 prd or Pax eyg subfamily members in any of the *H. exemplaris* databases that we analyzed. We analyzed the genome assembly of a second tardigrade species—*R. varieornatus* [62] using reciprocal BLAST searches, but did not find matches for either of these Pax subfamily members.

### Expression pattern of *He*-*otd*

**Fig. 6:**
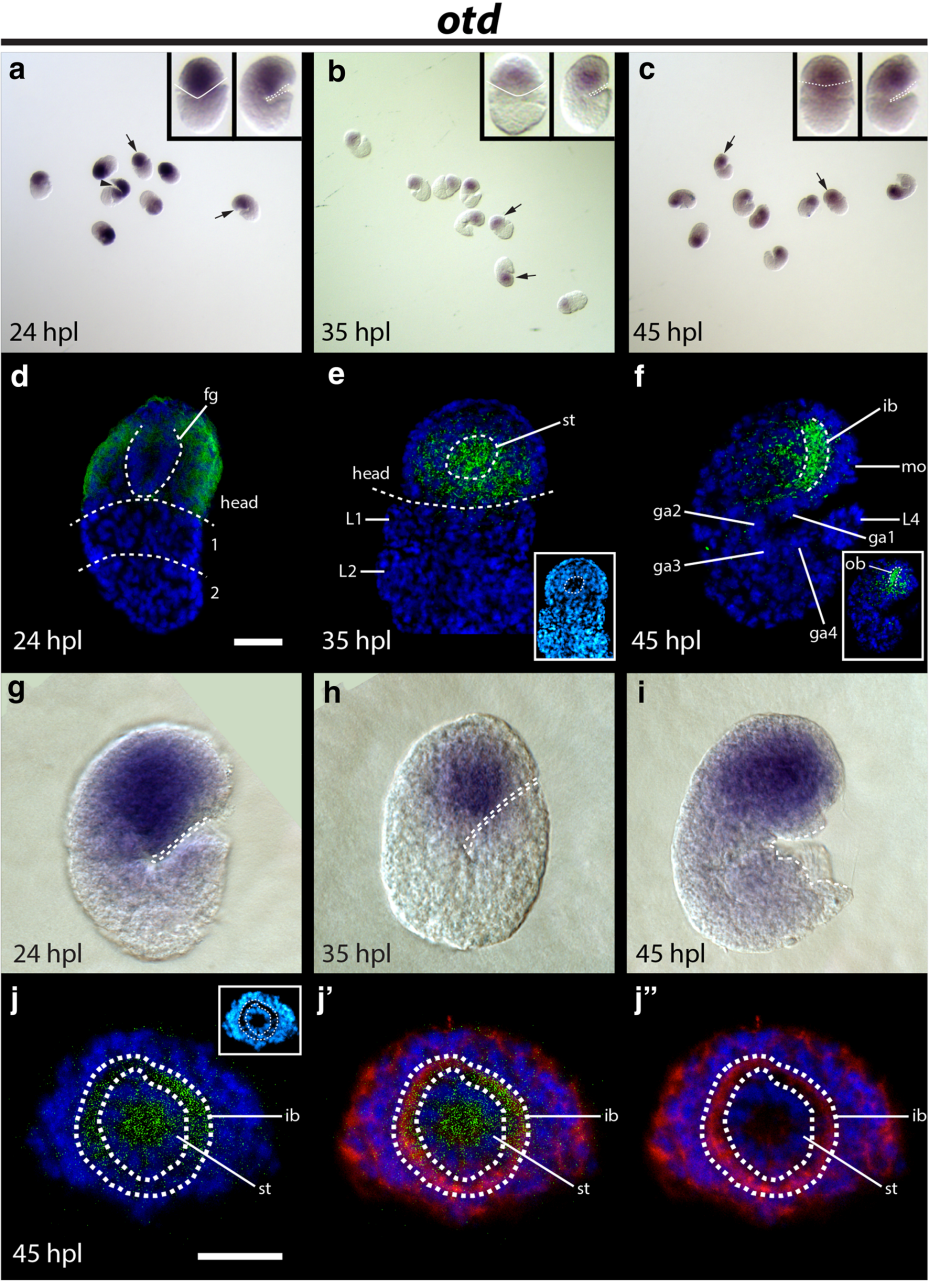
Embryonic expression patterns of *He*-*otd*. **a**–**c** DIC micrographs of several specimens from *He*-*otd* in situ hybridizations. Arrows point to specimens that are enlarged in inset panels. **a** The arrowhead points to damage to the head of a specimen; this cut does not represent the body axis. (Inset **a**–**c**) Specimens are facing forward in the left panels of (**a**, **b**). The solid line traces the space between the anterior and posterior ends of the specimens. In left inset panel in (**c**), the specimen is facing backward. The dashed line traces the boundary between the head and first trunk segment. In the right inset panels, specimens are facing right. In the right inset panel in (**b**), the image is mirrored. **d**–**f**, **j**–**j**′″) Confocal micrographs. *He*-*otd* expression is shown in green. DAPI labels nuclei (blue). **d** Dorsal view of a 24 hpl embryo. Dashed line in the head traces presumptive developing foregut. Dashed lines also trace boundaries between the head and first trunk segment and the first (1) and second (2) trunk segments. The scale bar equals 10 μm. **e** Ventral view of a 35 hpl embryo. Dashed line traces the boundary between the head and first trunk segment. In the head, the dashed line traces the nuclei of the developing stomodeal complex. (Inset) The same image using the cyan hot LUT in ImageJ to aid in visualization of embryo morphology. **f** Lateral view of a 45 hpl embryo. Dashed lines outline the inner brain region. (Inset) The same specimen, showing a more lateral view. The region where outer brain neuropil develops is outlined. **g**–**i** DIC micrographs of in situ hybridizations. A dashed line outlines the ventral surface of embryos. **j**–**j**″ Face-on view of the head of a 45 hpl embryo. β-Tubulin expression is colored red. Dashed lines trace the inner brain region. **j** Scale bar equals 10 μm. Inset shows DAPI using the cyan hot LUT in ImageJ to aid in visualization of embryo morphology. ga1–ga4, ganglion 1–ganglion 4; hpl, hours post-laying; ib, inner brain; L1–L4, leg1–leg4; fg, foregut; mo, mouth; ob, outer brain; st, stomodeal complex

At all stages that we analyzed, *He*-*otd* signal was strongly localized to the developing head (Fig. 6a–j). At 24 hpl, *He*-*otd* signal was detected broadly throughout the ectodermal layer of the head, extending to near the posterior border of the head (Fig. 6d) [45]. At 35 hpl, *He*-*otd* signal appeared strongest within the head, including in or near the developing stomodeal complex (st; Fig. 6e). At 45 hpl, we detected strong *He*-*otd* signal in the inner brain (ib) and outer brain (ob) neuropil (compare Fig. 6f to frames 11–27 of Additional file 5: Movie 1). *He*-*otd* signal colocalized with β-tubulin signal in the inner brain region (Fig. 6j). Strong *He*-*otd* signal was also detected in the developing stomodeal complex at 45 hpl (Fig. 6j).

### Identification of a *pax6* ortholog

**Fig. 7:**
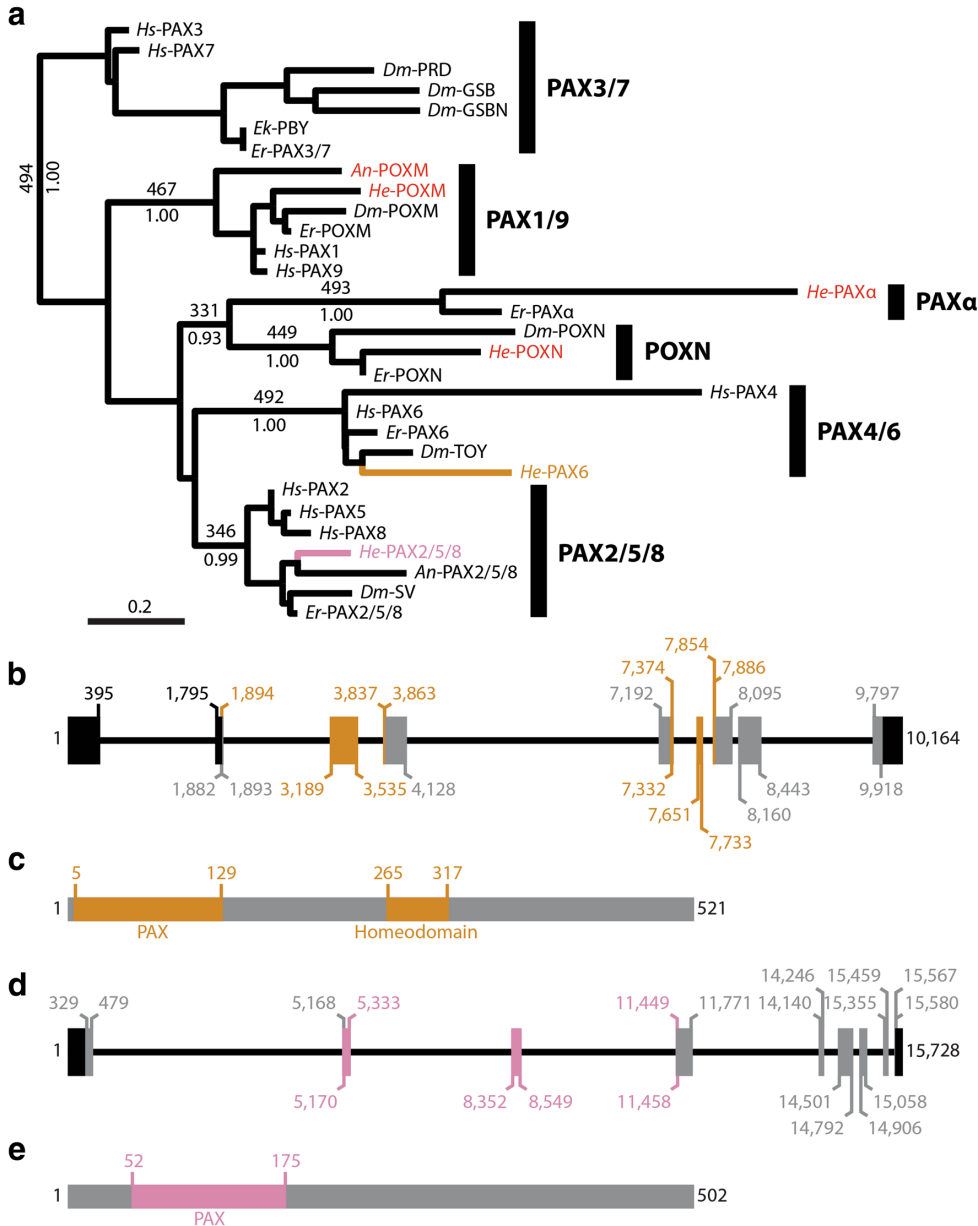
Phylogenetic and structural analysis of Pax genes. **a** Phylogenetic analysis of the Pax domain. Maximum likelihood tree topology is shown. Bootstrap support values are shown above select branches (out of 500 replicates) and posterior probabilities are shown below select branches. *He*-PAX6 is colored tan. *He*-PAX2/5/8 is colored pink. The names of other sequences from our analysis of *H. exemplaris* nucleotide data are colored red. **b** Structure of *He*-*pax6* gene. Relative to the coding strand, 5′ is to the left. Thick lines represent exons. Thin lines represent introns. Black exons represent UTR. Tan exons represent sequences that code for the PAX domain and the homeodomain. Gray exons represent other coding sequences. **c** Structure of the *He*-PAX6 protein. **d** Structure of *He*-*pax2/5/8* gene. The same labeling scheme that is used in panel b is used here, except that the PAX domain is colored pink. **e** Structure of the *He*-PAX2/5/8 protein. (b–e) Numbers represent nucleotide or amino acid positions (see Additional file 1: Table S1). *An*, anonymous sequence (see main text); *Dm*, *Drosophila melanogaster*; *Ek, Euperipatoides kanangrensis*; *Er*, *Euperipatoides rowelli*; *He*, *Hypsibius exemplaris*; *Hs*, *Homo sapiens*

PAX6 proteins generally contain a paired box domain (PAX) and a homeodomain [68, 69]. We identified a single *H. exemplaris* ortholog of *pax6* in our analysis of PRD Class homeodomains (Fig. 7a). *He*-PAX6 was nested within a well-supported monophyletic PAX6 clade in this tree (416/500, bootstrap support; 1.00 Bayesian posterior probability). We also performed phylogenetic analyses on a matrix that included 112 amino acids of the paired box domain (Additional file 4: FASTA alignments). This same gene was also nested within a monophyletic PAX6 clade in our analysis of PAX domains (Fig. 7a; Additional file 1: Table S1; Additional file 2: Table S2). Support for this clade was low in our PAX domain analysis, but we recovered good support for the more inclusive clade PAX4/6 (492/500, bootstrap support; 1.00 Bayesian posterior probability). A Pax domain and a homeodomain were the only conserved domains that we detected in *He*-PAX6 (Fig. 7b, c). We also detected an ortholog of *paxα* (Fig. 7a), a gene that is predicted to have been present in the nephrozoan ancestor, but independently lost in the chordate and *Drosophila* lineages [69]. We did not include PAX EYG proteins in our analysis of PAX domains because the *D. melanogaster* PAX EYG members—Eyegone and Twin of Eyegone—have highly divergent PAX domains, and we had already established that *H. exemplaris* was missing a *pax eyg* ortholog (Fig. 5a). As with our analysis of PRD Class homeodomains (Fig. 5a), we did not detect an *H. exemplaris* PAX3/7 member in our analysis of PAX domains (Fig. 7a).

### Expression pattern of *He*-*pax6*

**Fig. 8:**
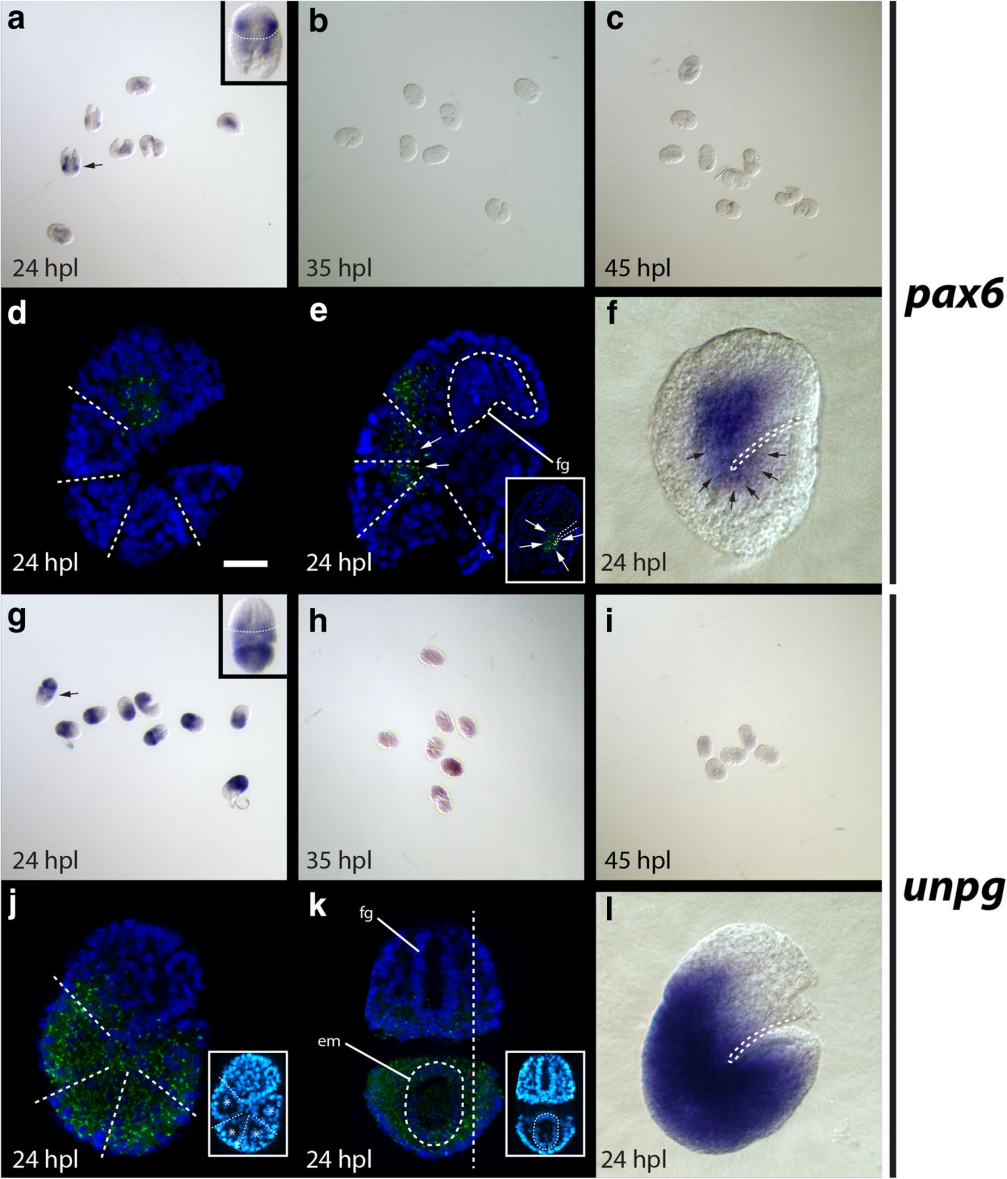
Embryonic expression patterns of *He*-*pax6* and *He*-*unpg*. **a**–**c**, **g**–**i** Fields of embryos at different developmental stages. **a**, **g** Arrows point to specimens that are enlarged in inset panels. Inset, **a**, **g** Anterior is toward the top. The backs of the specimens are facing forward. Dashed lines demarcate the approximate boundary between the head and first trunk segment. **d**, **e**, **j**, **k** Confocal micrographs. Gene expression is shown in green. DAPI labels nuclei (blue). **a**–**f** *He*-*pax6* expression detected with in situ hybridization. **d** Lateral view of a 24 hpl embryo. Dashed lines trace segment boundaries. The scale bar equals 10 μm. **e**, **f** Arrows point to gene expression in the ventral trunk region. **e** Oblique view of a 24 hpl embryo showing expression in the head and first two trunk segments. Dashed lines trace segment boundaries and outline the developing foregut. (Inset) A deeper focal plane showing the presumptive ventral neurectoderm of all trunk segments. **f** Lateral view of a 24 hpl embryo. The dashed line traces the ventral surface of the embryo. **g**–**l** *He*-*unpg* expression detected with in situ hybridization. **j** Lateral view of a 24 hpl embryo. Dashed lines trace segment boundaries. (Inset) The same specimen showing DAPI staining using the Cyan Hot LUT in ImageJ to aid in visualizing morphology. The asterisks denote the position of endomesodermal pouches. **k** Dorsoventral mounted 24 hpl embryo. The dashed oval in the trunk (bottom) outlines the endomesodermal cell layer of an oblique transverse section through the third trunk segment. The vertical dashed line shows the approximate position of the focal plane shown in (**j**). (Inset) The same specimen showing DAPI staining using the Cyan Hot LUT in ImageJ to aid in visualizing morphology. **l** Lateral view of a 24 hpl embryo. em, endomesodermal cell layer; fg, developing foregut; hpl, hours post-laying

At 24 hpl, the in situ pattern was consistent between embryos, but signal intensity appeared variable (Fig. 8a), suggesting that there are dynamic changes in expression levels of *He*-*pax6* at this development stage. We did not detect *He*-*pax6* transcripts at 35 hpl or 45 hpl (Fig. 8b, c), when nervous system morphology is apparent and *He*-*elav* signal is strong (Fig. 2). At 24 hpl, *He*-*pax6* signal was detected in a relatively large lateral region of the outer ectoderm of the developing head (Fig. 8d–f). We did not detect *He*-*pax6* signal in the internal layer of cells in the developing head, which we predict give rise to the foregut (fg; Fig. 8e). In the developing trunk, we detected *He*-*pax6* signal in paired ventromedial domains in the ectodermal cell layer (Fig. 8e, f).

### Identification of an *unpg* ortholog

**Fig. 9:**
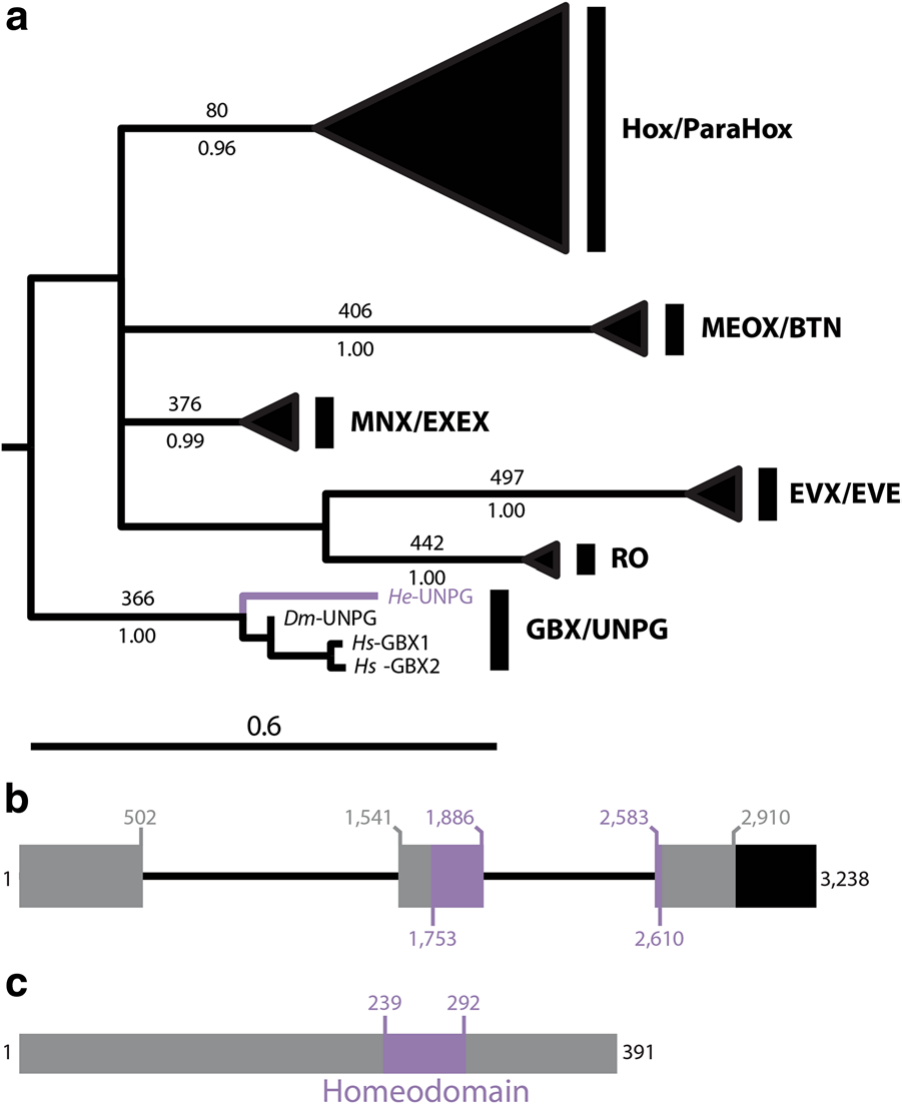
Phylogenetic and structural analysis of *He*-*unpg*. **a** Analysis of the ANTP Class genes based on an alignment of the homeodomain. Maximum likelihood tree topology is shown. The full ANTP Class phylogeny is shown in Additional file 7: Fig. S3. Bootstrap support values (out of 500 replicates) are shown above select branches. Posterior probabilities are shown below select branches. *He*-*unpg* is colored purple. **b** Structure of *He*-*unpg* gene. Relative to the coding strand, 5′ is to the left. Thick lines represent exons. Thin lines represent introns. Black exons represent UTR. Purple exons represent sequences that code for the homeodomain. Gray exons represent other protein coding sequences. **c** Structure of *He*-UNPG protein. **b**, **c** Numbers represent nucleotide or amino acid positions (see Additional file 1: Table S1). *Dm, Drosophila melanogaster*; *He*, *Hypsibius exemplaris*; *Hs*, *Homo sapiens*

*Gbx*/*unpg* codes for an ANTP class homeodomain protein. In order to identify an ortholog of *Gbx*/*unpg*, we looked for ANTP class homeobox genes in available *H. exemplaris* sequence data, using Ryan et al. [67] as a guide to choosing human and *D. melanogaster* ANTP class homeodomain proteins to use in our BLAST searches. We identified 35 candidate ANTP class homeobox genes with this method. We performed phylogenetic analyses on a matrix of 60 amino acids of the ANTP homeodomain from 167 sequences (Additional file 1: Table S1; Additional file 2: Table S2; Additional file 4: FASTA alignments). Of these candidates, one predicted protein sequence was most closely related to a monophyletic group of GBX/UNPG proteins (Fig. 9a; Additional file 7: Fig. S3). Together with the GBX/UNPG clade, this sequence formed a monophyletic group with good support (366/500, bootstrap support; 1.00 Bayesian posterior probability). Although it is not nested within the clade of previously identified GBX/UNPG sequences, it is a reciprocal best blast hit to both *Homo sapiens* and *D. melanogaster* GBX/UNPG sequences. Like human GBX1 and GBX2 and *D. melanogaster* UNPG, the homeodomain is the only conserved domain in the candidate GBX/UNPG sequence (Fig. 9b, c). We refer to this gene as *He*-*unpg*, since it is referred to as *unpg* in onychophorans [70] and flies [10].

### Expression pattern of *He*-*unpg*

We detected strong *He*-*unpg* signal at 24 hpl (Fig. 8g). *He*-*unpg* signal at 35 hpl and 45 hpl was either weak or absent (Fig. 8h, i), suggesting that it does not play an important role in later stages of nervous system development. At 24 hpl, *He*-*unpg* signal was detected broadly throughout the trunk (Fig. 8j–l). *He*-*unpg* signal was mostly absent in the developing head, but was detected in a relatively small dorsoposterior domain (Fig. 8j). Signal was detected most strongly between the nuclei of the ectodermal layer and the nuclei of the endomesodermal layer (em), but was still detectible within the endomesodermal layer (Fig. 8k).

### Identification of a *pax2/5/8* ortholog

We identified two candidate *pax2/5/8* orthologs in the initial BLAST search analysis of our genome assembly [49]. One of these candidates was found in all data sets except the transcriptome assemblies from Yoshida et al. [52] and Levin et al. [53]. We refer to this sequence *He*-*pax2/5/8*. The second candidate sequence was not identified in any other database that we analyzed (Additional file 2: Table S2). We were unable to amplify this sequence from genomic DNA using nested PCR (Additional file 3: Fig. S1). Furthermore, this predicted gene sequence is located on a small scaffold of just 2867 nucleotides in our genome assembly. It is the only predicated gene sequence on this scaffold. Therefore, we predict that this sequence represents a contaminant in our genome assembly. We refer to this sequence as anonymous *pax2/5/8* (*An*-*pax2/5/8*). We detected another PAX sequence in our genome assembly [49] that was not found in any other database. This predicted sequence was recovered in a highly supported PAX1/9 clade in our phylogenetic analysis (Fig. 7a). We were unable to amplify this gene from genomic DNA using nested PCR (Additional file 3: Fig. S1). Therefore, we refer to this sequence as anonymous *poxm* (*An*-*poxm*).

We analyzed a matrix of Pax genes that included 112 amino acids (Additional file 4: FASTA alignments). *He*-PAX2/5/8 and *An*-PAX2/5/8 were both nested within a monophyletic PAX2/5/8 clade in our analysis of PAX domains (Fig. 7a), supporting our assignment of these sequences to the *pax2/5/8* subfamily. The PAX domain was the only domain that we detected in *He*-*pax2/5/8* (Fig. 7d, e). Likewise, a PAX domain was the only conserved domain detected in CD-Search analyses of the *D. melanogaster* and *Euperipatoides rowelli* (Onychophora) *pax2/5/8* orthologs that we used in our phylogenetic analyses.

### Expression pattern of *He*-*pax2/5/8*

**Fig. 10:**
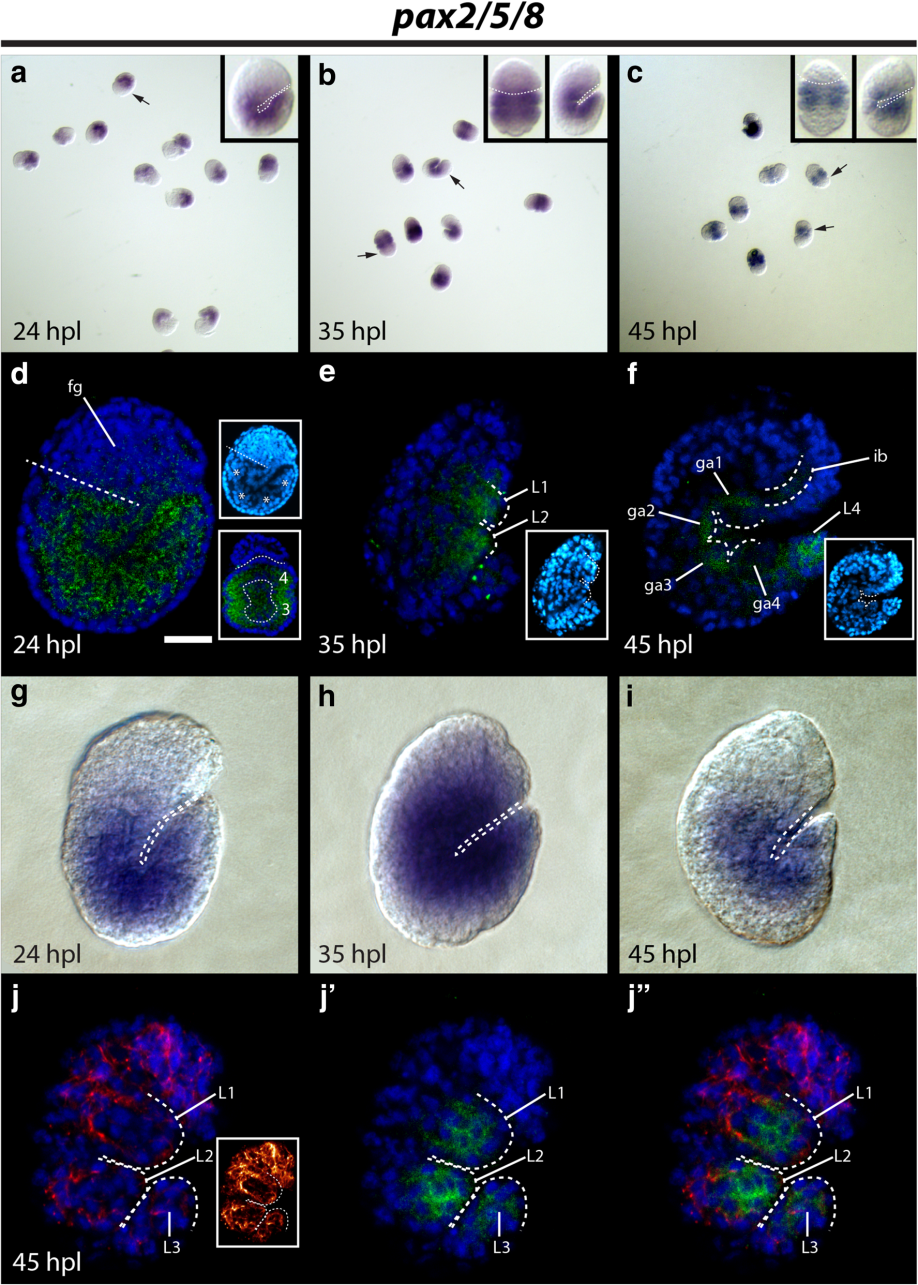
Embryonic expression patterns of *He*-*pax2/5/8*. **a**–**c** DIC micrographs of several specimens from *He*-*pax2/5/8* in situ hybridizations. Arrows point to specimens that are enlarged in inset panels. Inset, **a** A laterally mounted embryo. The dashed line traces the ventral surface. Inset, **b**, **c** The left panels show embryos facing backward. The dashed line traces the approximate boundary of the head and first trunk segment. The right panels show embryos facing right. In the inset panel in (**c**), the image is mirrored. **d**–**f**, **j**–**j**″ Confocal micrographs. *He*-*pax2/5/8* expression is shown in green. DAPI labels nuclei (blue). **d** Lateral view of a 24 hpl embryo. The dashed line traces the boundary between the head and first trunk segment. The scale bar equals 10 μm. (Inset, top) The same specimen shown with DAPI signal visualized with the Cyan Hot LUT in ImageJ to help visualize morphology. The asterisks denote the position of endomesodermal pouches. (Inset, bottom) A dorsoventrally mounted 24 hpl embryo. The upper dashed line traces the boundary between the head (above) and the posterior end (below) of the embryo. An oblique coronal plane of the third (3) and fourth (4) trunk segments is shown. The lower dashed line traces the nuclei of the endomesodermal cell layer of the third and fourth trunk segments. **e** Lateral view of a 35 hpl embryo. Dashed lines trace the developing first and second legs. **f** Lateral view of a 45 hpl embryo. Dashed lines outline the inner brain region and the trunk ganglia. (Inset) The same specimen, with DAPI signal viewed with the Cyan Hot LUT in ImageJ to better visualize morphology. **g**–**i** DIC micrographs of in situ hybridizations. A dashed line outlines the ventral surface of embryos. **j**–**j**″ Lateral view of a 45 hpl embryo. This is the same specimen that is shown in (**f**). Dashed lines trace the first three legs. β-Tubulin expression (red) is shown to help visualize leg morphology. Inset, **j** β-tubulin expression visualized with the Glow LUT in ImageJ to help visualize the nervous system. fg, foregut; ga1–ga4, ganglion 1–ganglion 4; hpl, hours post-laying; ib, inner brain; L1–L4, leg1–leg4

*He*-*pax2/5/8* signal was detected at all developmental stages of our study (Fig. 10a–c). Strong *He*-*pax2/5/8* signal was detected in the developing trunk at all stages investigated (Fig. 10a–i). At 24 hpl, strong *He*-*pax2/5/8* signal was detected between the ectodermal layer and endomesodermal layer and within the endomesodermal layer of the developing trunk (Fig. 10d). At 35 hpl, *He*-*pax2/5/8* signal was detected in the developing leg buds (L1–L2; Fig. 10e). At 45 hpl, *He*-*pax2/5/8* signal was detected in the developing trunk ganglia (ga1–ga4; Fig. 10f) and legs (L4; Fig. 10c; L1–L3; Fig. 10j).

## Discussion

An earlier reconstruction of the nervous system of the ancient nephrozoan ancestor relied on developmental data from just two species—mice and flies [10]. Analyses of additional taxa are required to more confidently resolve the evolution of nervous systems in Nephrozoa [1, 32, 33]. *D. melanogaster* is part of the highly diverse lineage Euarthropoda. Euarthropoda is part of a larger lineage called Panarthropoda that includes Tardigrada and Onychophora. Like euarthropods, both tardigrades [37, 41, 42, 47, 71–74] and onychophorans [75–79] exhibit centralized nervous systems and complex brains. Reconstructing the evolution of nervous systems in Panarthropoda requires consideration of nervous system development in representatives of all three panarthropod lineages. According to two phylogenomic studies of Panarthropoda that carefully controlled for long branch attraction artifacts, Tardigrada is the outgroup of a euarthropod + onychophoran lineage [34, 36], which makes the tardigrade lineage, especially important for reconstructing nervous system evolution in Panarthropoda. Here, we compare the results of our study to studies of euarthropods and onychophorans to reconstruct nervous system evolution in Panarthropoda.

### Tardigrades have a unipartite brain

**Fig. 11:**
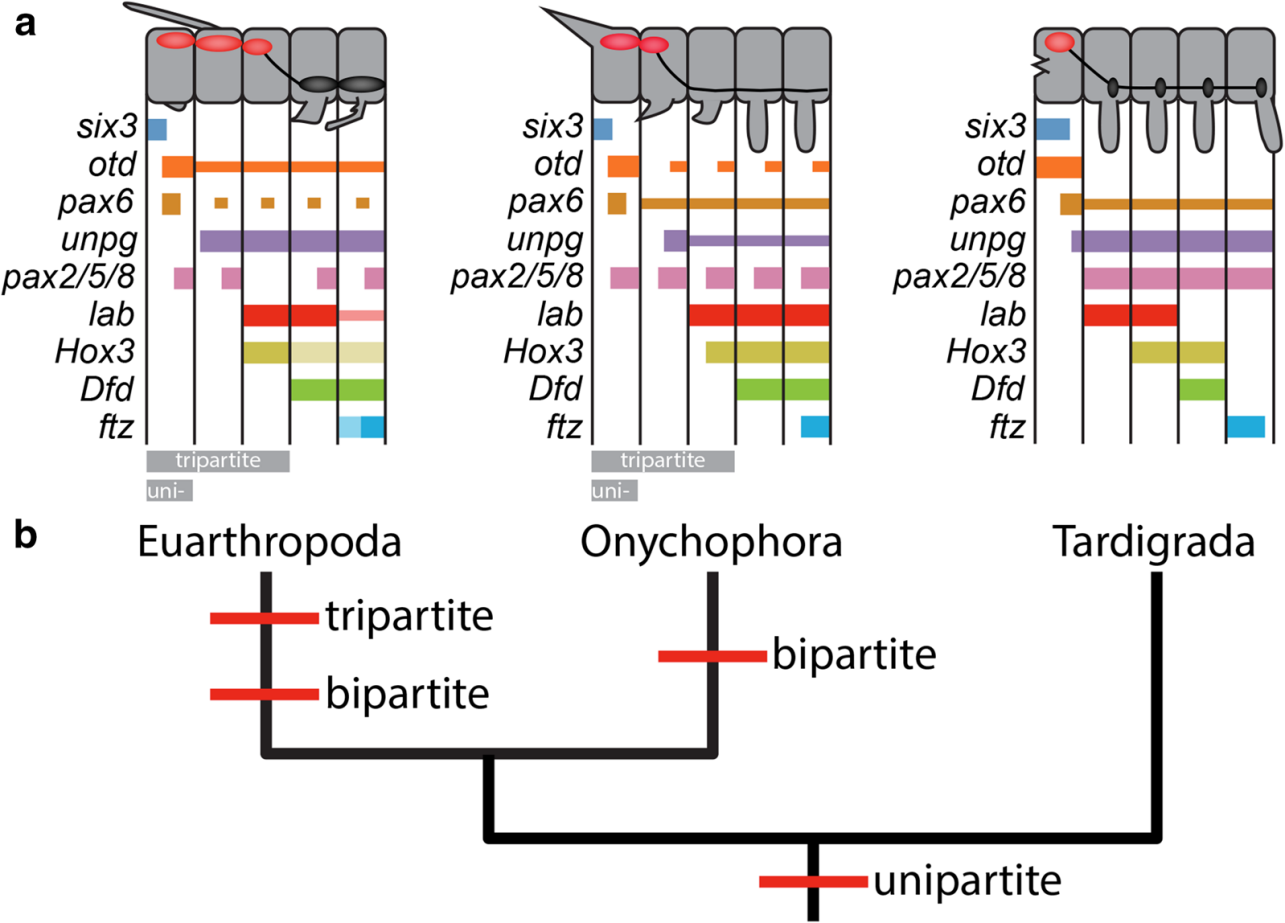
The evolution of panarthropod brains. **a** Models depicting gene expression domains in euarthropods (left), onychophorans (center), and tardigrades (right). In the anatomical models, red ovals represent brain segments, black ovals represent ganglia, and black lines that connect to ovals represent connectives. In the models of gene expression domains, thin bars represent reduced expression or more restricted expression. Light bars represent expression domains that have been identified in some species, but not others. Gray bars labeled “tripartite” and “unipartite” underscore the segments that are homologous to the tardigrade head under these competing hypotheses. **b** Model for the evolution of panarthropod brains based on developmental, morphological, and paleontological data. The phylogeny is based on Campbell et al. [34] and Rota-Stabelli et al. [36]

To determine whether tardigrades have a tripartite or unipartite brain, it is necessary to determine how the head of tardigrades—the brain housing unit—relates to segments of other panarthropods. Previous studies have compared expression patterns of developmental genes to homologize segments between chelicerates and mandibulates [80–82] and between euarthropods and onychophorans [83, 84]. The expression patterns of developmental genes in anterior segments are remarkably similar across the euarthropod/onychophoran clade (Fig. 11a). *Six3* is expressed in the anterior part of the first segment [16, 85]. *Otd* is expressed broadly in a more posterior domain of the first segment and in a more restricted mid-ventral region of more posterior segments [82, 85–89]. *Pax6* is expressed in a large lateral domain in the first segment and in more restricted ventral domains in more posterior segments [17, 19, 85, 89–91]. The anterior expression border of *unpg* is in the second segment in *D. melanogaster* [10, 92] and onychophorans [70]. *Pax2/5/8* is expressed segmentally in *D. melanogaster* [10] and onychophorans [90], with the anteriormost expression domain located in the first segment. The anterior expression borders of *labial* (*lab*), *proboscipedia* (*pb*), and *Hox3* are in the third segment, *Deformed* (*Dfd*) in the fourth segment, and *fushi tarazu* (*ftz*) in the fifth segment in both euarthropods [93, 94] and onychophorans [83, 84]. Results of these studies indicate that—in terms of homology—anterior segments of euarthropods and onychophorans align one to one in anterior–posterior order.

The tripartite brain hypothesis suggests that the tardigrade head is homologous to the first three segments of euarthropods and onychophorans [38]. This hypothesis predicts that genes that are expressed in the first three segments of euarthropods and onychophorans should be expressed in the head of tardigrades. Our analyses of gene expression patterns in *H. exemplaris* contradict the tripartite hypothesis. The tripartite hypothesis predicts that *six3* should only be expressed in the anterior part of the first segment of a three-segment head (Fig. 11a). However, *He*-*six3* is expressed broadly in the head (Fig. 4), rather than being restricted to a small anterior domain. The tripartite hypothesis predicts that broad *otd* expression should be restricted to the first segment of a three-segment head (Fig. 11a). By contrast, broad expression of *He*-*otd* continues to the posterior border of the head (Fig. 6d). The same contradiction applies for *pax6* (Fig. 7a, b). The tripartite hypothesis predicts that both *labial* and *Hox3* should be expressed in the tardigrade head (Fig. 11a). In actuality, neither of these genes is expressed in the head of tardigrades [45]. Furthermore, this hypothesis predicts that the anterior expression border of *Dfd* and *ftz* should lie in the first and second trunk segment, respectively, in tardigrades (Fig. 11a). However, the anterior expression borders of these genes lie in the third and fourth trunk segments in tardigrades [45].

The unipartite hypothesis suggests that the tardigrade head is homologous to just the first segment of euarthropods and onychophorans [38]. In contrast to the tripartite brain hypothesis, the unipartite brain hypothesis correctly predicts expression patterns of several developmental genes in *H. exemplaris.* This hypothesis predicts that *six3*, *otd*, and *pax6* should be expressed broadly in the head of tardigrades (Fig. 11a), as is the case (Figs. 4, 6, 8a–c), although *He*-*six3* is even more broadly expressed than predicted (see below). Furthermore, this hypothesis correctly predicts the anterior expression domains of *Hox3*, *Dfd*, and *ftz* (Fig. 11a).

While the expression patterns of some genes support the unipartite hypothesis, expression patterns of *He*-*unpg*, *He*-*lab*, and *He*-*pax2/5/8* are not predicted by either the tripartite hypothesis or the unipartite hypothesis. The tripartite hypothesis predicts that anterior border of *unpg* expression should lie in the middle of the tardigrade head (Fig. 11a), a position that would correspond to the middle segment of a three-segment head. The unipartite hypothesis predicts that the anterior border of *unpg* should lie in the first trunk segment of tardigrades (Fig. 11a). However, the anterior border of *He*-*unpg* expression is in a posterior region of the developing head (Fig. 8j). Although this result is not predicted by either hypothesis, to our knowledge, in Panarthropoda, *unpg* expression has only been investigated in *D. melanogaster* and the onychophoran *Euperipatoides kanangrensis*. Even between these species, there appears to be variation in the exact position of the anterior border of *unpg* expression. Therefore, *unpg* expression should be investigated in additional panarthropod representatives to determine whether the anterior expression boundary of *He*-*unpg* is atypical. Concerning *He*-*lab*, the tripartite brain hypothesis predicts that the anterior expression boundary of this gene should be in the tardigrade head, in a region that corresponds to the third brain segment of euarthropods (Fig. 11a). The unipartite hypothesis predicts that the anterior expression boundary of *lab* should lie in the second trunk segment in tardigrades (Fig. 11a). In actuality, the anterior boundary of *He*-*lab* expression is in the first trunk segment [45]. If the unipartite hypothesis is correct, as suggested by the expression domains of several other genes (see above), then the anterior expression boundary of *lab* must have expanded into a more anterior segment in the tardigrade lineage, or retracted into a more posterior segment in the lineage leading to Euarthropoda and Onychophora.

Lastly, the tripartite hypothesis predicts that there should be two or three segmental expression domains of *pax2/5/8* in the tardigrade head, based on the expression patterns of this gene in *D. melanogaster* and onychophorans (Fig. 11a). The unipartite hypothesis predicts that *He*-*pax2/5/8* should exhibit a single segmental expression domain in the head (Fig. 11a). Matching neither hypothesis, strong *He*-*pax2/5/8* expression is restricted to the developing trunk (Fig. 10). In the case of *He*-*pax2/5/8*, comparisons to more distantly related animals reveal clues about the composition of the tardigrade brain. In most non-panarthropod animals that have been investigated, the anterior border of *pax2/5/8* abuts the posterior border of *otx/otd* expression [30]. In *H. exemplaris*, the anteriormost border of strong *pax2/5/8* expression closely aligns with the posterior border of strong *He*-*otd* expression where the developing ventral nerve cord meets the developing brain (compare Fig. 10c to Fig. 6f). Therefore, concerning the discrepancy between the anteriormost expression domain of *pax2/5/8* of *H. exemplaris* and other panarthropods, *H. exemplaris* most likely retains the ancestral condition. In sum, the weight of evidence supports the unipartite hypothesis for the composition of the tardigrade brain. Changes in expression domains of developmental genes have previously been implicated in the diversification of animal body plans [95–97], raising the possibility that changes in the expression domains of *lab*, *unpg*, and *pax2/5/8* played a role in the diversification of panarthropod body plans.

### *Six3* and *otd* patterns overlap extensively in the developing brain of *H. exemplaris*

Across Nephrozoa, expression and function of *six3* and *otd*/*otx* overlap minimally during development of the anterior nervous system—the brain of many animals [16]. *Six3* is expressed in the anteriormost median domain of the body axis of nephrozoans [29, 85, 98], where it is thought to play an ancient conserved role in specifying neurosecretory cells [16]. *Otx*/*otd* is typically expressed in a more posterolateral part of the developing brain, where it regulates eye development [16, 99–101]. In contrast to other animals, *He*-*six3* appeared to be expressed broadly across the developing head, including in lateral regions (Fig. 4), rather than being restricted to an anteromedial domain. It appeared that expression of *He*-*six3* and *He*-*otd* broadly overlapped in *H. exemplaris*, rather than being restricted to nearly non-overlapping expression domains, as seen in most other animals [16]. In fact, we detected expression of both genes in the developing inner brain region and stomodeal complex (Figs. 4e, f, j; 6e, f, j). Furthermore, in the outer parts of the head, strong *He*-*otd* signal was restricted to brain neuropil (Fig. 6f, inset), while *He*-*six3* was more broadly expressed (Fig. 4e, f). Comparing our results to a previous study of euarthropods, onychophorans, and annelids [16], it appears that *six3* expression expanded from an anteromedial domain into more posterior and lateral regions of the developing brain in the tardigrade lineage, after this lineage diverged from other animals. Determining where in tardigrade phylogeny expansion of *six3* evolved will require studies of additional tardigrade species, including species within Heterotardigrada—the most distant tardigrade relatives of *H. exemplaris*. Additionally, the exact location of the cell bodies where *otd* and *six3* are expressed in tardigrades would further illuminate the composition of the tardigrade brain.

### *Pax6* and *unpg* exhibit unique temporal dynamics

The canonical nervous system patterning genes that we investigated in this study all exhibited strong expression during the earliest stage that we investigated, 24 hpl. Of these genes, only two—*He*-*pax6* and *He*-*ungp*—were not expressed strongly at the later stages that we investigated (Fig. 8b, c, h, i). Orthologs of the canonical nervous system patterning genes, including *pax6* and *unpg* orthologs, continue to be expressed during development of onychophorans and euarthropods after appendages and the central nervous system are apparent [10, 16–19, 70, 82, 85–92], stages that are presumably later than the stages when *pax6* and *unpg* are expressed in *H. exemplaris*, since legs and the nervous system are not morphologically visible at 24 hpl in this species. While it is likely that *pax6* and *ungp* play roles in setting up the general regionalized pattern of the *H. exemplaris* nervous system, our results suggest that—unlike in onychophorans and euarthropods—these genes do not play later roles in nervous system patterning, such as specifying or maintaining neural cell types [3]. However, it is possible that these genes are expressed at later stages at a low level that is difficult to detect with our in situ hybridization method, but biologically significant. Testing this possibility will require establishment of new methods of visualizing gene expression in *H. exemplaris* and functional data for these genes.

### The evolution of the panarthropod Pax family

A recent analysis of Pax family genes that included transcriptome data from *H. exemplaris* and the onychophoran *E. rowelli* revealed a highly supported gene clade that was referred to as *paxα* [90]. Based on this study, it appears that the nephrozoan ancestor had at least seven Pax gene subfamilies [69]: (1) Pax alpha, (2) Pax eyg, (3) Pox neuro, (4) Pax4/6/10, (5) Pax2/5/8, (6) Pax3/7 prd, and (7) Pax1/9 meso. We identified Pax family genes in two analyses—an analysis of PRD Class homeodomains (Fig. 5), which are present in some Pax gene subfamilies [69, 102], and an analysis of PAX domains (Fig. 7). Our analyses suggest that *H. exemplaris* possesses single orthologs of each Pax gene subfamily except Pax eyg and Pax3/7. We were surprised to not find a *pax3/7* ortholog in any nucleotide database for *H. exemplaris*, since an ortholog of this gene is strongly predicted to have been present in the last common ancestor of Nephrozoa and is present in non-tardigrade panarthropods [69, 90, 102]. Additionally, we did not detect a *pax3/7* ortholog in the genome of the tardigrade *R. varieornatus.* Our results suggest that *pax3/7* was deleted somewhere in the tardigrade lineage after it split from the lineage leading to euarthropods and onychophorans, but before the divergence of the *H. exemplaris* and *R. varieornatus* lineages.

The conclusion that a Pax eyg ortholog was present in the nephrozoan last common ancestor is a relatively recent idea. This idea stems from a phylogenetic analysis of Pax family genes that recovered a clade of insect *pax eyg* genes and *pax eyg* candidates from sea urchins and hemichordates [103]. However, there does not appear to be a *pax eyg* ortholog in either tardigrades or onychophorans [90]. To our knowledge, *pax eyg* orthologs have not been identified in any animals outside of sea urchins, hemichordates, and Holometabola. Therefore, we predict that *pax eyg* evolved in the lineage leading to holometabolous insects, after this lineage split from other insects. In this view, previously identified *pax eyg* orthologs in sea urchins and hemichordates evolved independently from the *pax eyg* gene of Holometabola. The nomenclature of Pax genes should be modified to reflect the fact that the previously identified sequences referred to as *pax eyg* are not directly orthologous across Nephrozoa.

## Conclusion

The tripartite brain hypothesis suggests that the forebrain, midbrain, and hindbrain of vertebrates are directly homologous to the proto-, deuto-, and tritocerebral brain segments of flies. Therefore, this hypothesis suggests that the ancestor of vertebrates and flies—the last common ancestor of Nephrozoa—also exhibited a tripartite brain [2, 10, 12–15]. Based on this hypothesis, other nephrozoans should also exhibit tripartite brains. While all euarthropods are generally interpreted as possessing tripartite brains [39], developmental [83] and morphological [77] data suggest that Onychophora—the sister lineage of Euarthropoda [34, 36]—is characterized by a bipartite brain. Additionally, several stem representatives of both onychophorans and euarthropods exhibited unipartite brains [39, 104]. A comprehensive model of panarthropod brain evolution, based on investigations of extant and extinct representatives of Panarthropoda, presents a more parsimonious view of panarthropod brain evolution compared to the tripartite hypothesis [39, 104]. Under this model, the last common ancestor of Euarthropoda and Onychophora is predicted to have possessed a unipartite brain homologous to the protocerebrum of modern euarthropods. More complex brains evolved independently along the onycophoran and euarthropod lineages, according to models that include data from fossils [39, 104]. In the onychophoran lineage, the central nervous system of the second segment fused to the protocerebral brain, forming a bipartite brain. In the euarthropod lineage, the central nervous system of the second segment fused to the ancestral protocerebral brain, giving rise to the deutocerebral brain segment. The central nervous system of the third segment fused to the deutocerebrum, giving rise to the tritocerebral brain segment. Conceivably, tardigrades could have also independently evolved a multipartite brain—a possibility that draws support from the fact that the evolution of small body size is often associated with the fusion of segmental ganglia [105]. However, our analyses of brain pattering genes in *H. exemplaris* suggest that tardigrades possess a unipartite brain. Even if the tardigrade brain is currently unipartite, we need to consider the possibility that the tardigrade brain was secondarily simplified from a multipartite brain during the evolution of the tardigrade lineage. Although we cannot completely rule out this possibility, it is a less parsimonious view of brain evolution. Our results place the origin of the unipartite brain in the stem lineage leading to Panarthropoda and suggest that tardigrades retain the ancestral protocerebral-grade brain [39, 104]. This model of brain evolution is inconsistent with an ancestral tripartite nephrozoan brain.

Although the ancestor of Panarthropoda most likely exhibited a unipartite brain, it is clear based on comparisons between *H. exemplaris* and other panarthropods that its nervous system patterning genes exhibited regionalized expression patterns. Our study supports the model in which regionalized expression patterns of vertebrate and fly brain patterning genes evolved in a nephrozoan ancestor that did not possess a tripartite brain and that these regionalized patterns were co-opted independently for the evolution of tripartite brains in the vertebrate and euarthropod lineages [1, 24, 25, 29, 30].

## Additional files

**Additional file 1: Table S1.** Sources for *H. exemplaris* sequences. Identification numbers that are in bold refer to the sequences that were used to build the gene models and protein models that are depicted in figures. For *Hd-unpg*, we used two transcriptome sequences to build the genome model shown in Fig. 9b because the 5′ end of comp91475_c0 was not found in any scaffold that included *He-unpg* coding sequence. bHd01897.1 only included coding sequence, so we did not diagram 5′ UTR in Fig. 9b.

**Additional file 2: Table S2.** Sources for sequences used in phylogenetic analyses. For non-*H. exemplaris* sequences, GenBank accession numbers are provided. For *H. exemplaris* sequences, the source of the publically available genome or transcriptome assembly and the sequence identification number to access the sequence are provided. Three sequences were detected in our genome assembly [49], but no other assemblies—*An*-ELAV, *An*-PAX2/5/8, and *An-*POXM. For these sequences, we provide annotations.

**Additional file 3: Fig. S1.** Support for genome assembly contamination. (a, b) Numbers refer to product size (ladder) or expected product size. (a) Gel containing PCR results prepared with *H. exemplaris* genome as a template and primers for *An*-*elav*, *An*-*pax2/5/8*, and *An*-*poxm*. The outer primer pairs are referred to as F1, R1. The inner primer pairs are referred to as F2, R2. *He-actin* was used as a positive control. (b) Gel containing PCR results that used F1/R1 product of (a) as template and F2/R2 primers.

**Additional file 4: FASTA alignments.** Amino acid matrices that were analyzed in phylogenetic analyses.

**Additional file 5: Movie 1.** The central nervous system of an *H. exemplaris* embryo. The movie shows a laterally mounted 45 hpl embryo. Anterior is toward the top. The ventral side is facing toward the right. Nuclei are stained with DAPI (blue). The nervous system is stained with a fluorescent secondary antibody bound to a β-tubulin antibody (red). The movie was produced from 35 slices of a *Z*-series collected on a confocal laser scanning microscope. Slice 8 shows a neurite cluster that extends from the outer brain region (ob) to the first trunk ganglion. In the region between the first trunk ganglion and the head, this neurite cluster is referred to as the outer connective [47, 64]. In slices 10–17, the neurite cluster widens and more clearly represents brain neuropil. In slice 18, the region where the outer brain neuropil and the inner brain neuropil (ib) meet to give rise to the dorsal brain neuropil (dnp) is visible [64]. The inner brain neuropil extends from the dorsal position to a ventral position within the head (slices 18–28). At a ventral position within the head, the inner brain neuropil gives rise to the inner connective, which connects to the first trunk ganglion [47, 64]. The preoral brain commissure (prc), which connects the left and right inner brain regions above the mouth, is visible in slices 24–35 [64]. The post oral brain commissure, which connects the left and right inner brain regions below the mouth, is visible in slices 30–35 [64]. The right legs (L1–L4), trunk ganglia (ga1–ga4), and right ganglion connective (cn) are also visible.

**Additional file 6: Fig. S2.** Phylogeny of PRD Class genes. Maximum likelihood tree topology is shown. Bootstrap support values, out of 500 replicates, are shown above select branches, and Bayesian posterior probabilities are shown below these branches. *He-*OTD is colored orange. The names of other sequences from our analysis of *H. exemplaris* nucleotide data are colored red. Black vertical bars demarcate PRD gene families.

**Additional file 7: Fig. S3.** Phylogeny of ANTP Class genes. Maximum likelihood tree topology is shown. Bootstrap support values, out of 500 replicates, are shown above select branches, and Bayesian posterior probabilities are shown below these branches. *He-*UNPP is colored purple. The names of other sequences from our analysis of *H. exemplaris* nucleotide data are colored red. Black vertical bars demarcate ANTP gene families. Gray vertical bars indicate cases where it is unclear whether a *H. exemplaris* sequence belongs to a particular ANTP gene family, due to low phylogenetic support. *An*, anonymous sequence (see main text); *Ct*, *Capitella teleta*; *Dm*, *Drosophila melanogaster*; *He*, *Hypsibius exemplaris*; *Hs*, *Homo sapiens*.
