# Supplementary figures and images for: Analyses of nervous system patterning genes in the tardigrade Hypsibius exemplaris illuminate the evolution of panarthropod brains
Source: EvoDevo. 2018 Jul 30;9:19. doi: 10.1186/s13227-018-0106-1 (PMC6065069; doi:10.1186/s13227-018-0106-1)

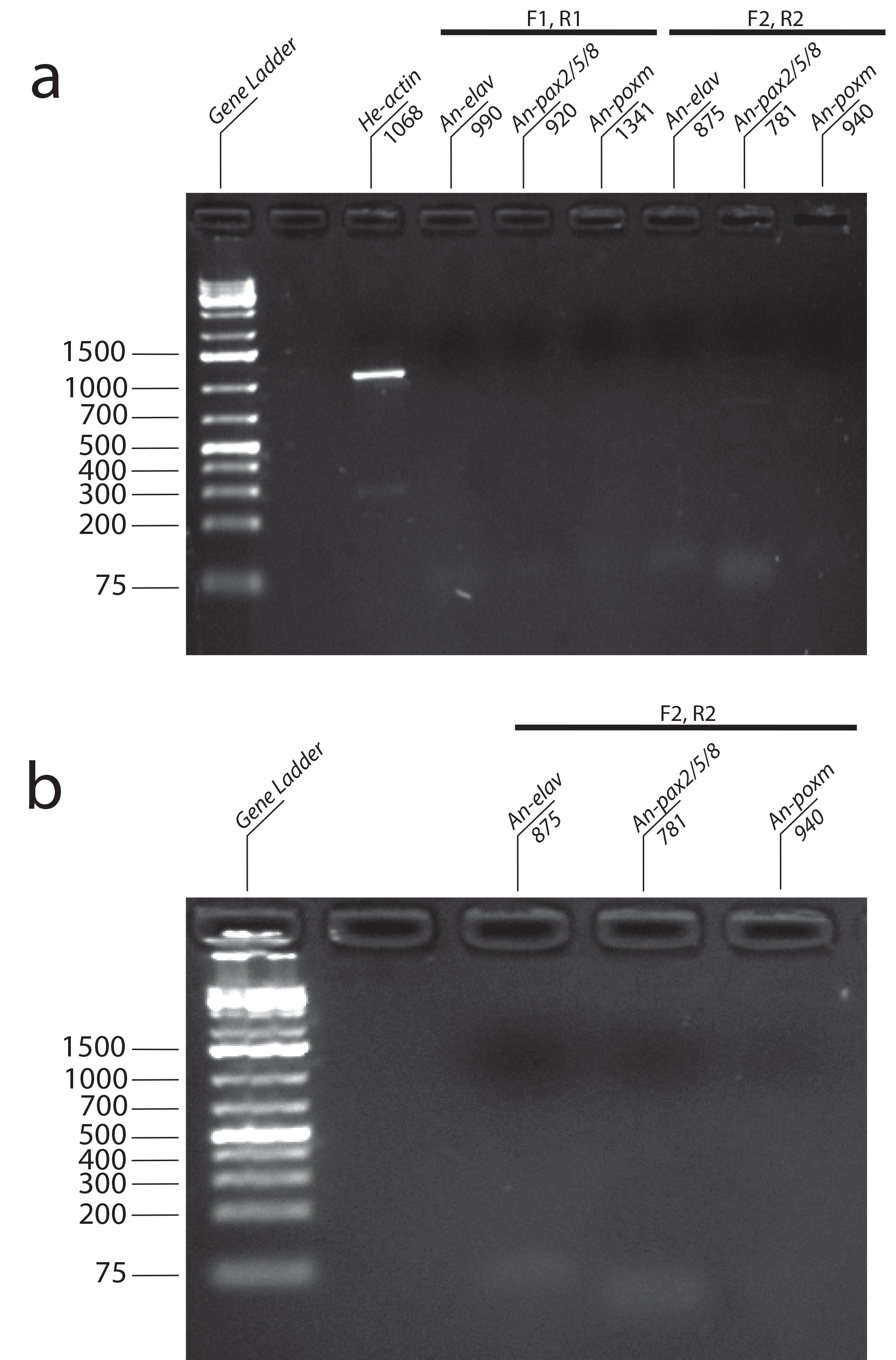

Supplement: Supplementary file 3 — Additional file 3: Fig. S1. Support for genome assembly contamination. (a, b) Numbers refer to product size (ladder) or expected product size. (a) Gel containing PCR results prepared with H. exemplaris genome as a template and primers for An-elav, An-pax2/5/8, and An-poxm. The outer primer pairs are referred to as F1, R1. The inner primer pairs are referred to as F2, R2. He-actin was used as a positive control. (b) Gel containing PCR results that used F1/R1 product of (a) as template and F2/R2 primers. [file 13227_2018_106_MOESM3_ESM.tif]

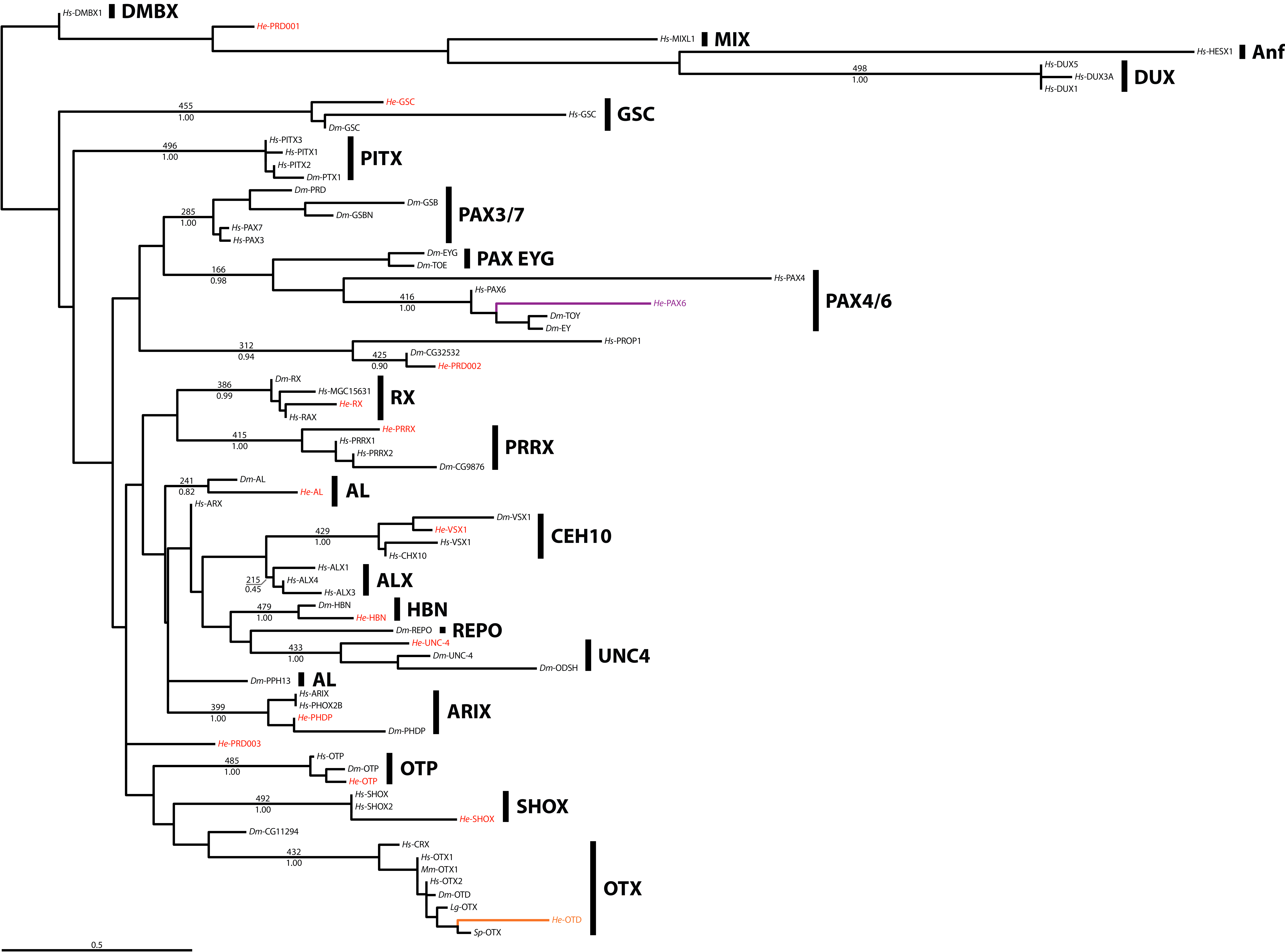

Supplement: Supplementary file 6 — Additional file 6: Fig. S2. Phylogeny of PRD Class genes. Maximum likelihood tree topology is shown. Bootstrap support values, out of 500 replicates, are shown above select branches, and Bayesian posterior probabilities are shown below these branches. He-OTD is colored orange. The names of other sequences from our analysis of H. exemplaris nucleotide data are colored red. Black vertical bars demarcate PRD gene families. [file 13227_2018_106_MOESM6_ESM.tif]
